# Molecular genetics of maternally-controlled cell divisions

**DOI:** 10.1371/journal.pgen.1008652

**Published:** 2020-04-08

**Authors:** Elliott W. Abrams, Ricardo Fuentes, Florence L. Marlow, Manami Kobayashi, Hong Zhang, Sumei Lu, Lee Kapp, Shai R. Joseph, Amy Kugath, Tripti Gupta, Virginia Lemon, Greg Runke, Amanda A. Amodeo, Nadine L. Vastenhouw, Mary C. Mullins

**Affiliations:** 1 Department of Cell and Developmental Biology, University of Pennsylvania Perelman School of Medicine, Philadelphia, Pennsylvania, United States of America; 2 Department of Biology, Purchase College, The State University of New York, Purchase, New York, United States of America; 3 Max Planck Institute of Molecular Cell Biology and Genetics, Dresden, Germany; 4 Lewis-Sigler Institute for Integrative Genomics, Princeton University, Princeton, New Jersey, United States of America; HudsonAlpha Institute for Biotechnology, UNITED STATES

## Abstract

Forward genetic screens remain at the forefront of biology as an unbiased approach for discovering and elucidating gene function at the organismal and molecular level. Past mutagenesis screens targeting maternal-effect genes identified a broad spectrum of phenotypes ranging from defects in oocyte development to embryonic patterning. However, earlier vertebrate screens did not reach saturation, anticipated classes of phenotypes were not uncovered, and technological limitations made it difficult to pinpoint the causal gene. In this study, we performed a chemically-induced maternal-effect mutagenesis screen in zebrafish and identified eight distinct mutants specifically affecting the cleavage stage of development and one cleavage stage mutant that is also male sterile. The cleavage-stage phenotypes fell into three separate classes: developmental arrest proximal to the mid blastula transition (MBT), irregular cleavage, and cytokinesis mutants. We mapped each mutation to narrow genetic intervals and determined the molecular basis for two of the developmental arrest mutants, and a mutation causing male sterility and a maternal-effect mutant phenotype. One developmental arrest mutant gene encodes a maternal specific Stem Loop Binding Protein, which is required to maintain maternal histone levels. The other developmental arrest mutant encodes a maternal-specific subunit of the Minichromosome Maintenance Protein Complex, which is essential for maintaining normal chromosome integrity in the early blastomeres. Finally, we identify a hypomorphic allele of Polo-like kinase-1 (Plk-1), which results in a male sterile and maternal-effect phenotype. Collectively, these mutants expand our molecular-genetic understanding of the maternal regulation of early embryonic development in vertebrates.

## Author summary

The earliest stages of animal development are regulated by factors in the egg that are made during oogenesis and are required for the embryo to develop prior to genome activation of the embryo itself. Because eggs are large, the cells of the early embryo are large and so unique mechanisms act during these stages of development. To study these molecular-genetic processes in a vertebrate, we have chemically induced mutations in the zebrafish germline and screened for mutant mothers (maternal-effect mutants) with defects in these processes. We identified three distinct classes of mutations affecting cell division in the early embryo. One of these mutations also exhibits a male-sterile phenotype. We identify the mutated genes in three of these mutants. We expect the remaining mutant lines will serve as important tools for elucidating molecular mechanisms involved in cell organization and/or positioning during the cleavage stage, as well as mechanisms critical for proper patterning of the early embryo.

## Introduction

After fertilization the newly formed zygote undergoes cellular cleavages, which are under the control of maternally-supplied gene products. The duration of the cleavage stage varies among organisms and is punctuated by the maternal-to-zygotic transition (MZT), at which point major zygotic genome activation (ZGA) occurs (reviewed in[1, 2]). In zebrafish the MZT occurs during the 10th cell cycle [3], whereas in mouse MZT occurs relatively early at the two-cell stage. However, in other mammalian systems the MZT takes place at later stages of development, at the 4 to 8-cell transition in humans [4], and at the 8 to 16-cell transition in rabbit and sheep [5].

In some vertebrates, such as frogs and fish, the MZT coincides with the mid-blastula transition (MBT) [3, 6]. Prior to the MBT, cells divide synchronously and lack intervening gap phases during interphase of the cell cycle, allowing for more rapid cell division. In addition, cell cycle checkpoints are absent during the cleavage stage prior to the MBT [3]. At the MBT the cell cycle lengthens, becomes asynchronous and, along with widespread ZGA, cell migration is initiated and developmental processes such as gastrulation and epiboly ensue. Because genetic access to maternal gene functions in vertebrates remains challenging, the molecular underpinnings of genome stability, chromosomal architecture and cellular integrity during these rapid cell divisions devoid of checkpoints are still poorly understood.

The zebrafish has emerged as a valuable molecular-genetic model for identifying genes important for early vertebrate development. In the past, the majority of genetic screens performed in zebrafish have focused on targeting zygotic genes [7–9]. More recent mutagenesis screens have been designed to identify maternal genes acting during early embryonic development [10–13]. These initial screens have identified broad categories of maternal-effect mutations affecting processes ranging from pre-fertilization events, such as oocyte polarity [10, 14, 15], to processes occurring after the MBT, such as epiboly [13]. These screens were successful in establishing a collection of mutants with diverse phenotypic/genetic classes disrupting processes of importance to early vertebrate development. Moreover, these screens led to the discovery of new genes and novel roles for known genes in maternally-controlled processes [16–22]. However, it became clear that expected genes, such as cell cycle regulators, were not recovered, indicating that the screens had not reached saturation and that novel maternal regulators of cleavage-stage embryogenesis remained to be discovered.

To identify genes critical for cleavage-stage embryogenesis, we performed an ENU-induced maternal-effect mutagenesis screen in zebrafish. Here we report on two classes of mutants that disrupt the cleavage stage. The first class undergoes irregular cleavages prior to the MBT. The second class undergoes developmental arrest around the MZT and possesses varying degrees of nuclear/chromosomal defects. A third class of mutants identified in this screen, affecting cytokinesis, has been reported elsewhere [22]. During the course of our screen we also identified a male-sterile mutant, which we also report here. We mapped each mutation to relatively narrow genetic intervals. We positionally cloned the developmental arrest mutant *p10umal* and found that it encodes, Mini Chromosome Maintenance 3-like (Mcm3l), which is a maternal-specific subunit of the helicase complex acting in DNA synthesis licensing. In addition, we cloned a second developmental arrest mutant gene *screeching halt* (*srh*) identified in a previous screen [13] and found that it encodes Stem Loop Binding Protein 2 (SLBP2). SLBPs bind the 3’ UTR stem loop of replication-coupled histone mRNAs and are involved in histone metabolism during the S phase of the cell cycle [23]. We show that histone proteins are reduced during the cleavage stage in *srh* mutant embryos and that the *srh* arrest phenotype can be rescued by injecting whole histone protein, thus demonstrating that Slbp2 is required for maintaining sufficient histone levels during the cleavage stage of development. Our studies identify maternal-effect genes that function during the cleavage stage and, for two genes, provide molecular insight into maternally-controlled cell divisions during this period of development.

## Results and discussion

### Identification of cleavage-stage mutants

We performed a maternal-effect ENU mutagenesis screen in the zebrafish *Danio rerio* and identified nine mutants disrupting oocyte and/or egg development (prior to fertilization, manuscript in preparation) and nine mutants disrupting the cleavage stage of development (immediately following fertilization). One of the mutations from the latter group also causes a male sterile phenotype. The cleavage-stage mutants primarily fell into three classes: developmental arrest, irregular cleavage, and cytokinesis mutants (Table 1). The two cytokinesis mutants, *motley* (*mot*^*p01aiue*^) and *p04anua*, are described elsewhere [22]. In this study for simplicity we refer to the embryos obtained from homozygous mutant mothers as mutant embryos.

**Table 1:** Cleavage Stage Maternal-effect Mutants.

Class	Allele	Chr, map position^a^	Gene identity ^b^	Additional references ^c^
Developmental arrest	*srh*^*p18ad*^	21, z9233	*slbp2*	Wagner et al., 2004 Abrams et al., 2012
	*srh*^*sa12562*^	21	*slbp2*	
	*bmb*^*p22atuz*^	25, z1772	*brambleberry*	
	*p10umal*	20, z3824	*mcm3-like*	
	*sa1624*	20	*mcm3-like*	
Irregular cleavages	*dsy*^*p86batl*^	17, z4862	ND	
	*dul*^*p15uzat*^	20, z3211	ND	
	*mxp*^*p09batl*^	8, z7370	ND	
	*cld*^*p40atuza*^	15, z6985	ND	
	*p09ajug*	1, z11369	*polo-like kinase 1*	
Cytokinesis defects	*mot*^*p01aiue*^	23, z14967	*birc5b*	Nair et al., 2013 Nair et al., 2013
	*p04anua*	ND	ND	

(a) Chromosome number, closely linked marker; zebrafish genome assembly version 9 (Zv9) was used for linkage analysis to determine the map position of the mutant alleles.

(b) *slbp2 = stem loop binding protein 2*, *mcm3-like = mini-chromosome maintenance 3-like*, ND = not yet determined

(c) references outside of the present study.

**Fig 1:**
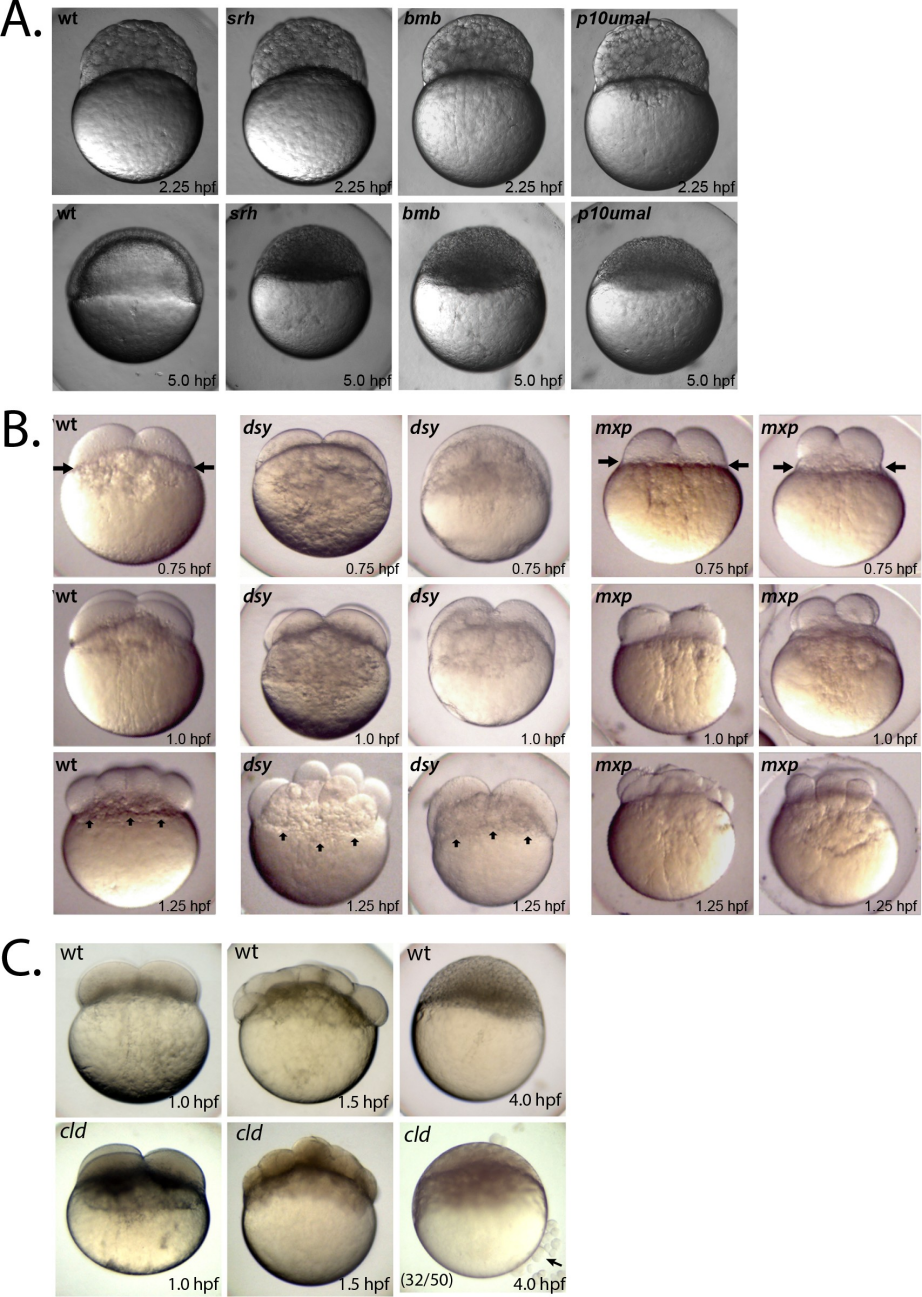
Maternal-effect cleavage-stage mutants. **A.** Developmental arrest mutants and wild-type at 2.25 hpf (top row) and at 5.0 hpf (bottom row). For each mutant the phenotype is 100% penetrant within a clutch and across mutant females. At least 50 embryos per mutant female were examined (n = 233 from four *srh* females, n = 854 from six *bmb* females, n = 335 from four *p10umal* females). **B.** Irregular cleavage mutants and wild type at 0.75 hpf (top row), 1.0 hpf (middle row) and 1.25 hpf (bottom row). Five of 6 *mxp* females produced embryos shown (n = 459), while one produced embryos with a more mild phenotype (n = 91). **C.** Wild-type (top row) and *cld* mutants (bottom row) at 1.0, 1.5, and 4.0 hpf. Penetrance of the cell sloughing phenotype (black arrow) at 4.0 hpf is indicated in lower left corner. Remaining embryos retain relatively normal, but dark, blastoderms and do not survive to 24 hpf.

We mapped each mutation to a chromosomal locus using bulk segregant analysis [24], demonstrating that each one corresponds to a distinct gene (Table 1). The two developmental arrest mutants, *brambleberry* (*bmb*^*p22atuz*^, [17]) and *p10umal*, failed to undergo epiboly (Fig 1A), arrested their development around the MBT and ultimately underwent lysis prior to 1-day post fertilization (dpf), similar to *screeching halt* (*srh*) mutants ([13]; Fig 1A). All three arrest mutant phenotypes are strictly maternal, as heterozygous females crossed to homozygous males yielded wild-type embryos with no obvious zygotic phenotype (S1 Table).

In the three irregular cleavage mutants, *mixed up* (*mxp*^*p09batl*^), *disarray* (*dsy*^*p86batl*^) and *p09ajug*, the early blastomeres formed irregularly spaced and sized cells, possibly due to asynchronous cell division (Figs 1B and 6A). In *dsy*^*p86batl*^ embryos the yolk appears to invade the overlying blastomeres (Fig 1B, vertical arrows) and in *mxp*^*p09batl*^ embryos the blastomeres are consistently reduced in size (Fig 1B, horizontal arrows). We identified another unique cleavage-stage mutant, *cloudy day* (*cld*^*p40atuza*^). Cleavage-stage mutant embryos of *cld*^*p40atuza*^ were comparable to wild type prior to the 1000-cell stage, except that they were much darker in appearance throughout the cleavage period (Fig 1C). In addition, beyond the 1000-cell stage, a significant percentage (64%) of blastula had cells sloughing off the blastoderm and the blastoderm itself lacked cell membranes (Fig 1C). Finally, one cleavage stage mutant, *dullahan* (*dul*^*p15uzat*^), frequently had an enlarged cytoplasmic domain below the blastoderm and at 24 hours post fertilization (hpf) displayed a ventralized phenotype (see below).

### Nuclear structure in *mxp* and *dsy* mutants

**Fig 2:**
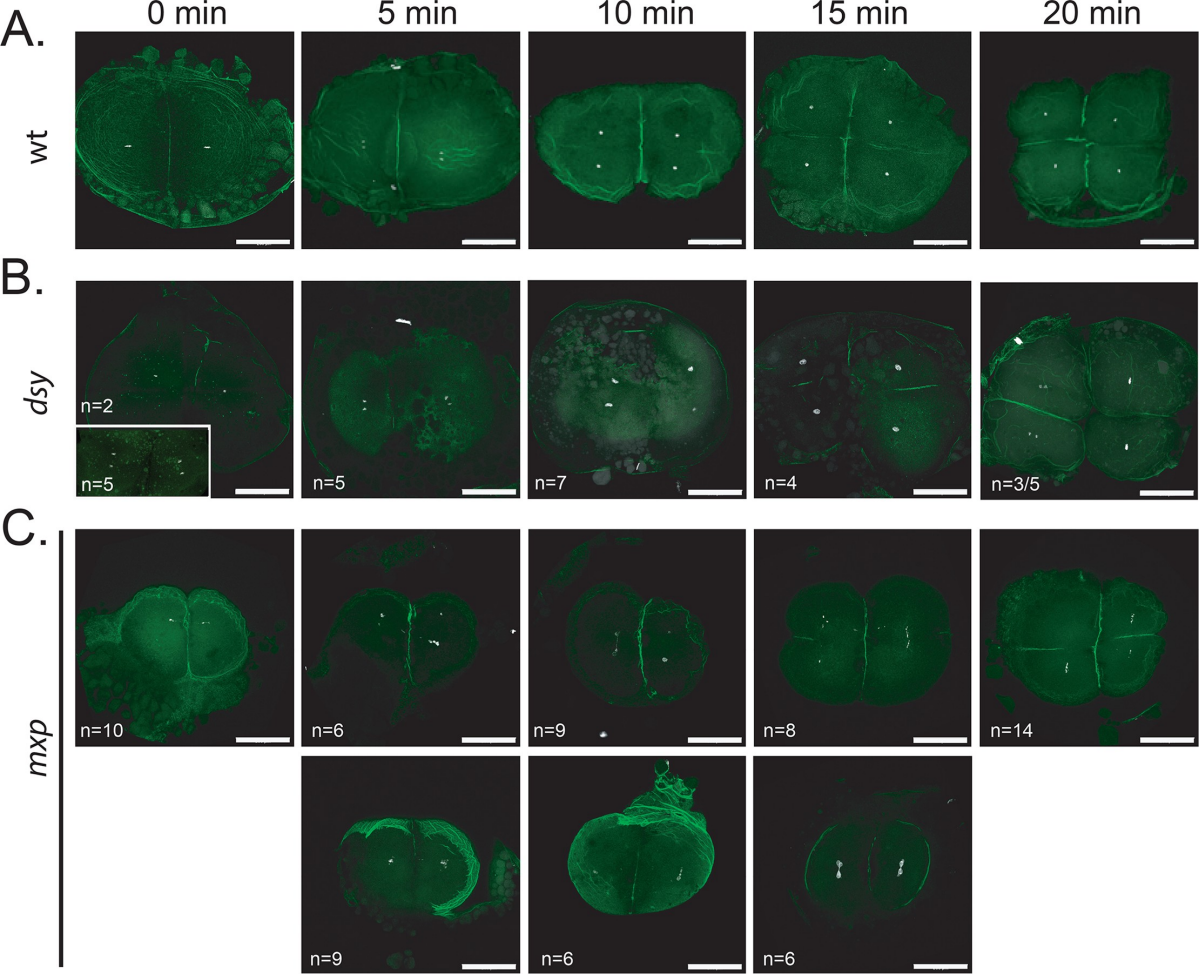
Examining nuclear integrity in *mixed up* and *disarray* embryos. **A.** Wild-type (TL), (**B**) *dsy* and (**C**) *mxp* embryos were fixed at 5-minute intervals spanning 20 minutes (corresponding to the 2 to 4 cell division) and stained with DAPI and phalloidin to mark the DNA and actin at the cell boundaries, respectively. **B**. Representative embryos (numbers indicated in the lower left corner) from a total of three *dsy* females. In some cases nuclear divisions were asynchronous (20 min) in embryos from *dsy* mutant mothers compared to wild type (A). **C**. Representative embryos (numbers indicated in the lower left corner) from a total of four *mxp* females. Embryos shown in the upper row underwent cell division timing similar to wild type in (A), whereas the embryos in the lower row were delayed. Scale bars = 200μm.

To examine nuclear integrity in the irregular cleavage mutants, we performed a time-course experiment spanning the 2- to 4-cell stages, staining the embryos with DAPI and for actin to mark the nuclei and cell boundaries, respectively. Nuclei appeared relatively normal in *dsy*^*p86batl*^ embryos (Fig 2B). In some cases there was evidence of asynchronous nuclear divisions (Fig 2B, 20 min). In comparison, nuclei in *mxp*^*p09batl*^ embryos appeared fragmented in each of the blastomeres (Fig 2C). In many cases, cell division was also delayed during this period (Fig 2C, bottom row) compared to wild type (Fig 2A). The molecular cloning of each corresponding gene will help clarify the nature of these defects and reveal important insight into the mechanisms involved in cell division timing during the cleavage stage of development.

### Nuclear division is disrupted in *p10umal* mutants

**Fig 3:**
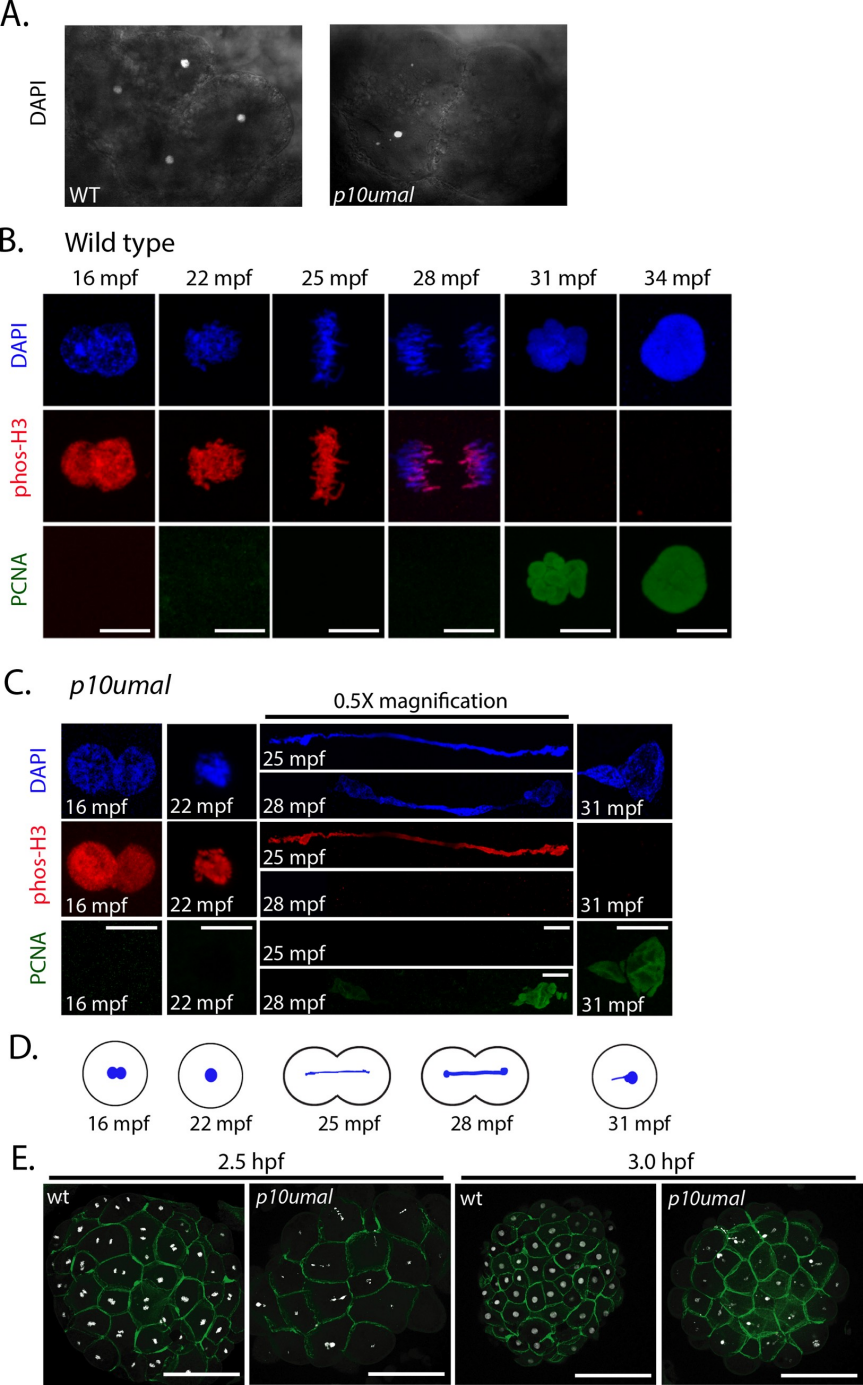
Nuclear division is disrupted in *p10umal* mutants. **A.** DAPI staining of wild-type and *p10umal* 8-cell stage embryos (n = 11). Note: only four of the 8-cells are in view. **B.** Wild-type and **C.** *p10umal* fertilization time courses (N = 3 females examined). Embryos were fixed at 16, 22, 25, 28, 31 and 34 mpf. A minimum of five embryos corresponding to each time point were examined (representative images are shown). Pronuclei (16 mpf) and the one-cell zygote (at 22–34 mpf) were stained with DAPI (blue), anti-phospho-histone H3 (red), and anti-PCNA (green). Scale bars = 10μm. The 25 and 28 mpf time points were digitally reduced by 0.5x. **D.** Schematic representation of the *p10umal* phenotype at the corresponding time points illustrating the typical DNA bridge between dividing cells. **E.** Wild type and *p10umal* mutants at 2.5 and 3.0 hpf stained with DAPI and phalloidin to mark DNA and the cell boundaries, respectively. A minimum of 3 and up to 6 embryos each from 3 different females were examined for each time point (representative images are shown).

Since the *p10umal* developmental arrest defect was nearly identical to the *bmb* phenotype and *bmb* mutants display multiple micronuclei during the cleavage stage [17], we examined the nuclei in early cleavage-stage *p10umal* mutants. In 8-cell *p10umal* embryos only one blastomere was typically DAPI-positive; however, minor DAPI signals could be detected in at least one additional blastomere (Fig 3A). These nuclei were smaller and fragmented compared to wild-type embryos. The remaining six blastomeres were DAPI-negative, indicating that they were anucleate. These data suggest that DNA synthesis and/or DNA segregation is defective in *p10umal* cleavage-stage embryos. Interestingly, it also shows that cytokinesis is programmed independently of mitosis during the cleavage stage, since cytokinesis continues although mitosis fails. This has also been previously reported for the *futile cycle* (*fue*) zebrafish maternal-effect mutant [12].

To determine when during development the *p10umal* defect is first evident, we performed a fertilization time course spanning pronuclear congression and the first mitotic division. We fixed embryos at 3-minute intervals starting at 10 minutes post fertilization (mpf). To precisely follow mitotic progression, embryos were stained with phospho-histone H3 (phos-H3) and PCNA to mark mitosis and interphase, respectively. Pronuclear congression and the first mitotic prophase appeared normal in *p10umal* embryos (compare Fig 3B and 3C, 16 and 22 mpf). However, at time points corresponding to the first metaphase and anaphase, individual chromosomes could not be detected in *p10umal* cells. Instead, phos-H3 and DAPI staining revealed DNA bridges that spanned and connected the dividing cells (Fig 3C, 25 and 28 mpf). Interestingly, despite these defects in DNA segregation, PCNA staining was detected at the appropriate timepoint of the cell cycle, consistent with the absence of cell cycle check points during the cleavage stage of embryogenesis. Thus, *p10umal* disrupts the first metaphase of embryogenesis.

We next examined the mid-blastula stage developmental arrest phenotype of *p10umal* mutant embryos at 2.5 and 3.0 hpf using phalloidin and DAPI staining. The *p10umal* cells in 3.0 hpf embryos appeared similar in size and shape to corresponding wild type embryos at 2.5 hpf, suggesting an approximate one cell cycle delay in the mutant (Fig 3E). Interestingly, most of the *p10umal* cells were either DAPI negative or contained small DAPI positive fragments. In addition, frequent DNA bridges were present spanning cells at both of these stages (Fig 3E). Thus, the early DNA segregation defects observed in *p10umal* mutants appeared to persist to these later stages.

### *p10umal* encodes minichromosome maintenance protein 3-like

**Fig 4:**
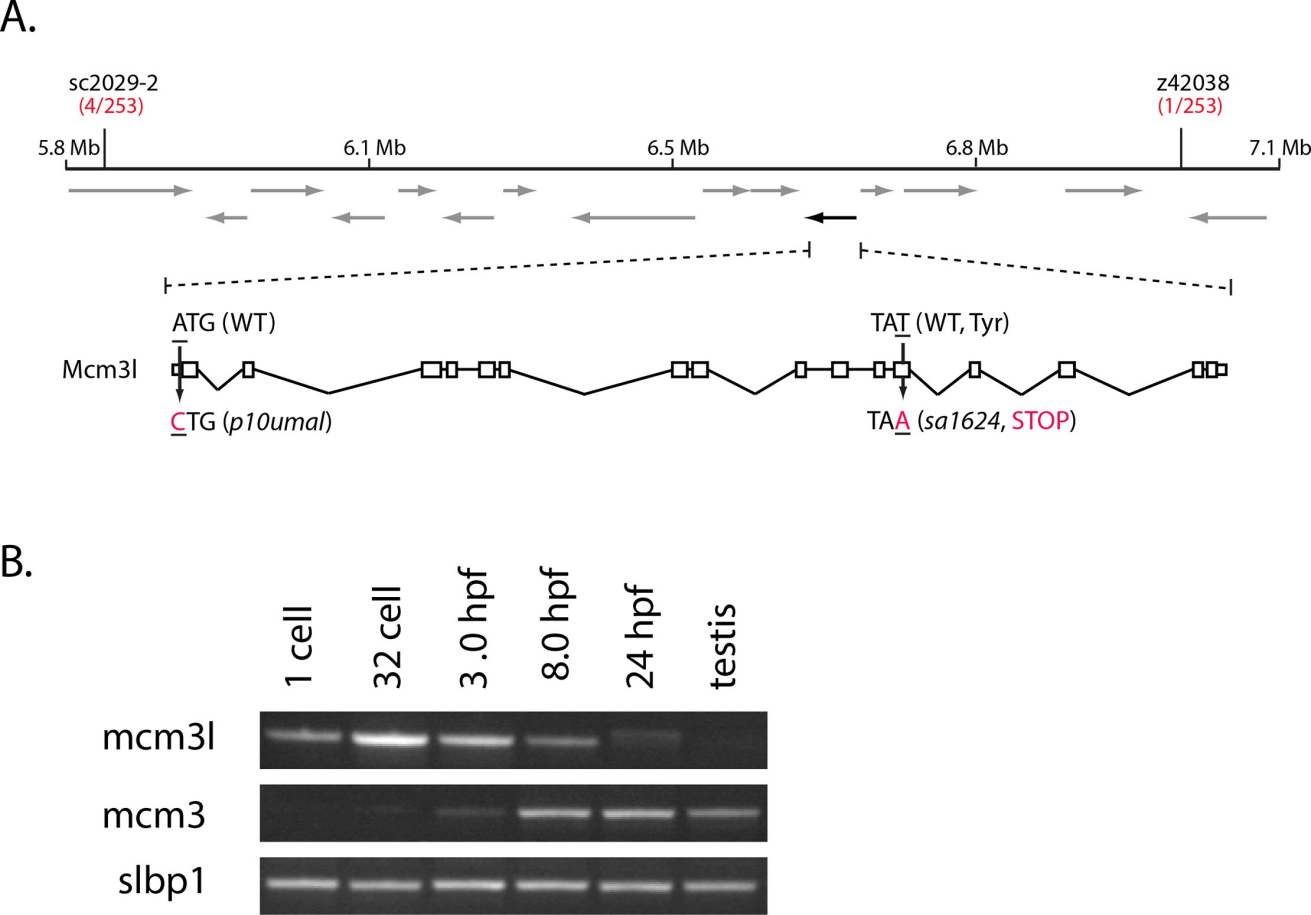
*p10umal* encodes *mcm3l*. **A.** *p10umal* maps to chromosome 20 within a 1.1 Mb interval flanked by sc2029-2 and z42038. This interval contains 14 predicted ORFs (arrows). The black arrow (on the reverse strand) corresponds to *mcm3l*. The genomic structure of *mcm3l* is shown with 5’ oriented to the left. Both alleles (*p10umal* and *sa1624*) are indicated. **B.** RT-PCR of *mcm3* and *mcm3l* in a developmental profile. Stage is indicated at the top and *slbp1* is used as a loading control.

To determine the molecular nature of the *p10umal* mutation, we positionally cloned the corresponding gene. By examining 253 meiotic recombination events, we mapped *p10umal* to a physical interval of 1.1 Mb on chromosome 20 (Fig 4A). This interval is flanked by simple sequence length polymorphic (SSLP) markers sc2029-2 and z43038 and contains 12 annotated genes and one predicted open reading frame (ORF) [25]. One gene, *minichromosome maintenance protein 3-like* (*mcm3l*) is highly related to *mcm3*, which is a component of the pre-initiation complex required for DNA replication found in all eukaryotes (reviewed in [26]). Sequence analysis of *mcm3l* ovary cDNA from *p10umal* fish revealed a missense mutation (A to C) in the ATG start codon (Fig 4A), changing the methionine to a leucine. Consequently, translation initiation is predicted to be disrupted such that translation is potentially initiated at the next downstream methionine (Met^37^), forming an N-terminally truncated protein (S1 Fig).

To determine if disrupting *mcm3l* function was responsible for the *p10umal* phenotype, we examined a mutant allele of *mcm3l* identified in the Zebrafish Mutation Project (ZMP) stock collection [27]. The *sa1624* allele contains a T to A change at nucleotide position 1793 of the *mcm3l* ORF. This mutation produces a premature stop codon within the ORF, resulting in a predicted truncation of Mcm3l at amino acid 591 of 807 total residues. Furthermore, we found that homozygous *mcm3l*^*sa1624*^ mutant females produced maternal-effect mutant embryos that arrested at a mid-blastula stage in development like *p10umal* mutant embryos and exhibited the same nuclear defect during cleavage. In addition, both *mcm3l* alleles were strictly recessive-maternal. Finally, the *sa1624* and *p10umal* mutations failed to complement each other in trans-heterozygous females, which produced the same maternal-effect embryonic defects as the single alleles (S2 Table). These results show that the maternal-effect defects observed in the *p10umal* and *sa1624* mutant embryos are caused by disruption of Mcm3l function.

In addition to *mcm3l*, the zebrafish genome contains a canonical *mcm3* gene, which is also located on chromosome 20 and is more homologous to mammalian Mcm3 [28]. RT-PCR revealed that *mcm3* expression begins at 3.0 hpf, corresponding to the onset of widespread zygotic transcription at the MBT, and persisted through 24 hpf (Fig 4B). Transcripts could also be detected in the adult testis (Fig 4B). In contrast, *mcm3l* mRNA expression was detected throughout the cleavage stage (Fig 4B, 1-cell and 32-cell stages, 3.0 hpf). Lower signal of *mcm3l* was detected at 8.0 hpf, and it was barely detectable at 24 hpf, and not detected in the adult testis (Fig 4B). These developmental profiles are consistent with *mcm3* and *mcm3l* expression studies previously reported in frogs and fish [28], and are consistent with *mcm3l* acting maternally, and *mcm3* functioning zygotically. These data suggest that Mcm3l is a cleavage-stage specific component of the Mcm hexamer that is required to initiate DNA replication. Thus, Mcm3l functions maternally during the cleavage stage, whereas Mcm3 functions zygotically throughout the rest of development and in adults.

Our *mcm3l* loss-of-function results demonstrate a specific maternal function for Mcm3l (homozygous males are completely normal). In *p10umal* embryos, DNA segregation is impaired during the first cell division (Fig 3C), a phenotype likely due to a defect in DNA synthesis. Interestingly, it has also been reported that the Mcm3 protein contains a functional nuclear localization signal, whereas maternal Mcm3l does not [28]. Whether Mcm3l has acquired specific functions during the cleavage stage possibly related to the increased rate of cell division and/or the lack of checkpoints specific to this period of development will require future rescue experiments of *p10umal* with a transgene of the canonical *mcm3*.

### Ventralized *dullahan* mutant embryos

**Fig 5:**
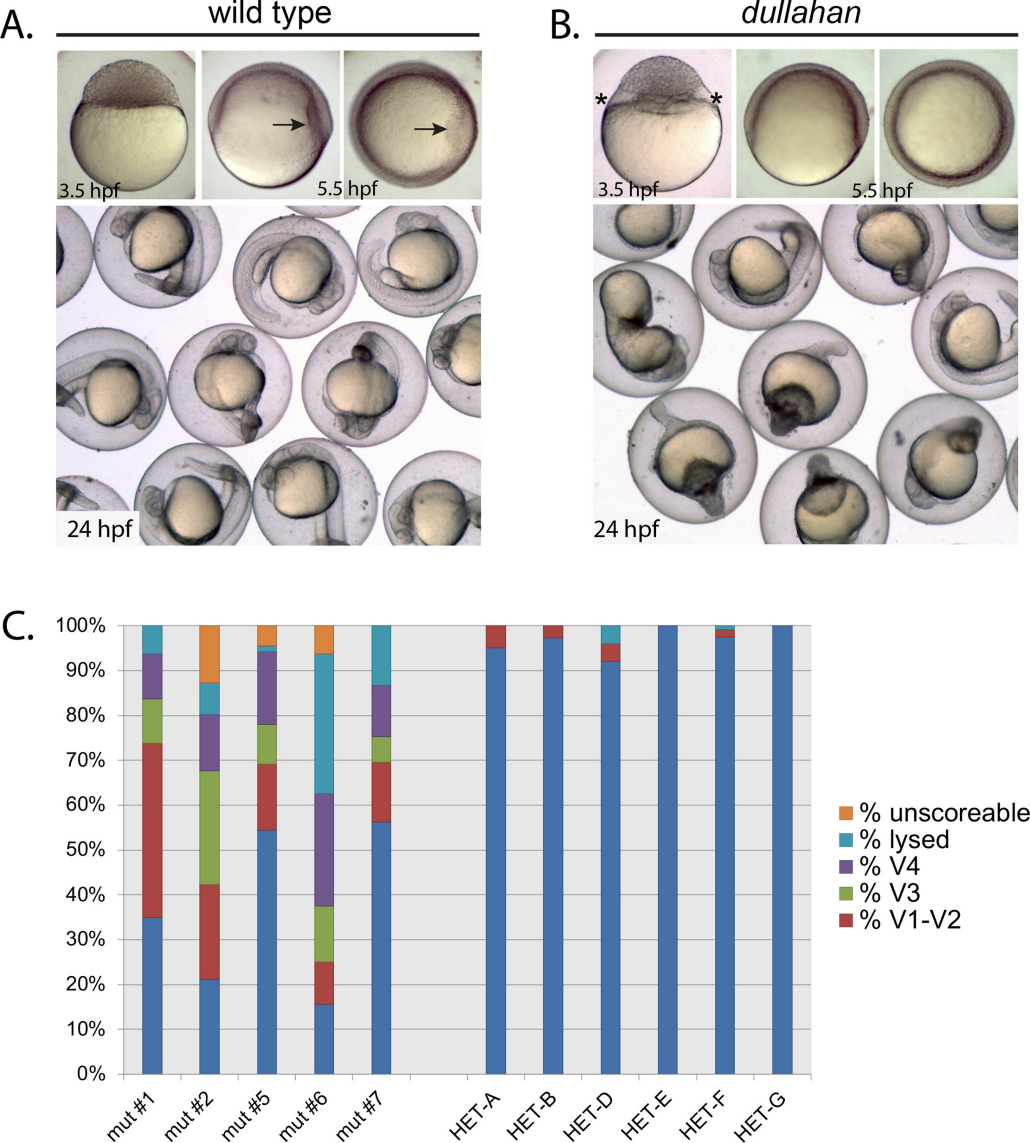
*dullahan* mutant phenotype. **A.** Wild-type and (**B**) *dul* embryos at 3.5, 5.5, and 24 hpf. Embryos in upper left and center panels are lateral views, and upper right panels are animal pole views. The enlarged cytoplasmic region between the yolk and blastomeres in the *dullahan* mutant at 3.5 hpf is noted with asterisks. The dorsal shield (arrow) is to the right in the 5.5 hpf wild-type embryo and absent in the mutant. **C.** Distribution of embryonic phenotypes from five *dul* females (left) compared to 6 siblings (right): mut #1 (n = 80), mut #2 (n = 71), mut #5 (n = 68), mut #6 (n = 32), mut #7 (n = 105), HET-A (n = 103), HET-B (n = 112), HET-C (n = 76), HET-D (n = 76), HET-A (n = 67), HET-A (n = 118), HET-G (n = 32). In each cross heterozygous or mutant females were crossed to wild-type (TL) males.

One cleavage stage mutant, *dullahan* (*dul*^*p15uzat*^), exhibited a clearing at the yolk cytoplasmic region underlying the blastoderm margin in most mid-blastula embryos (Compare Fig 5A and 5B, 3.5 hpf). Interestingly, in a majority of *dul*^*p15uzat*^ gastrula embryos the shield, which marks the prospective dorsal region and is the zebrafish equivalent of the dorsal organizer, was either significantly reduced or failed to form (Fig 5A and 5B, 5.5 hpf). By 24 hpf a large fraction of *dul* embryos were ventralized to varying degrees or had lysed (Fig 5A–5C).

**Fig 6:**
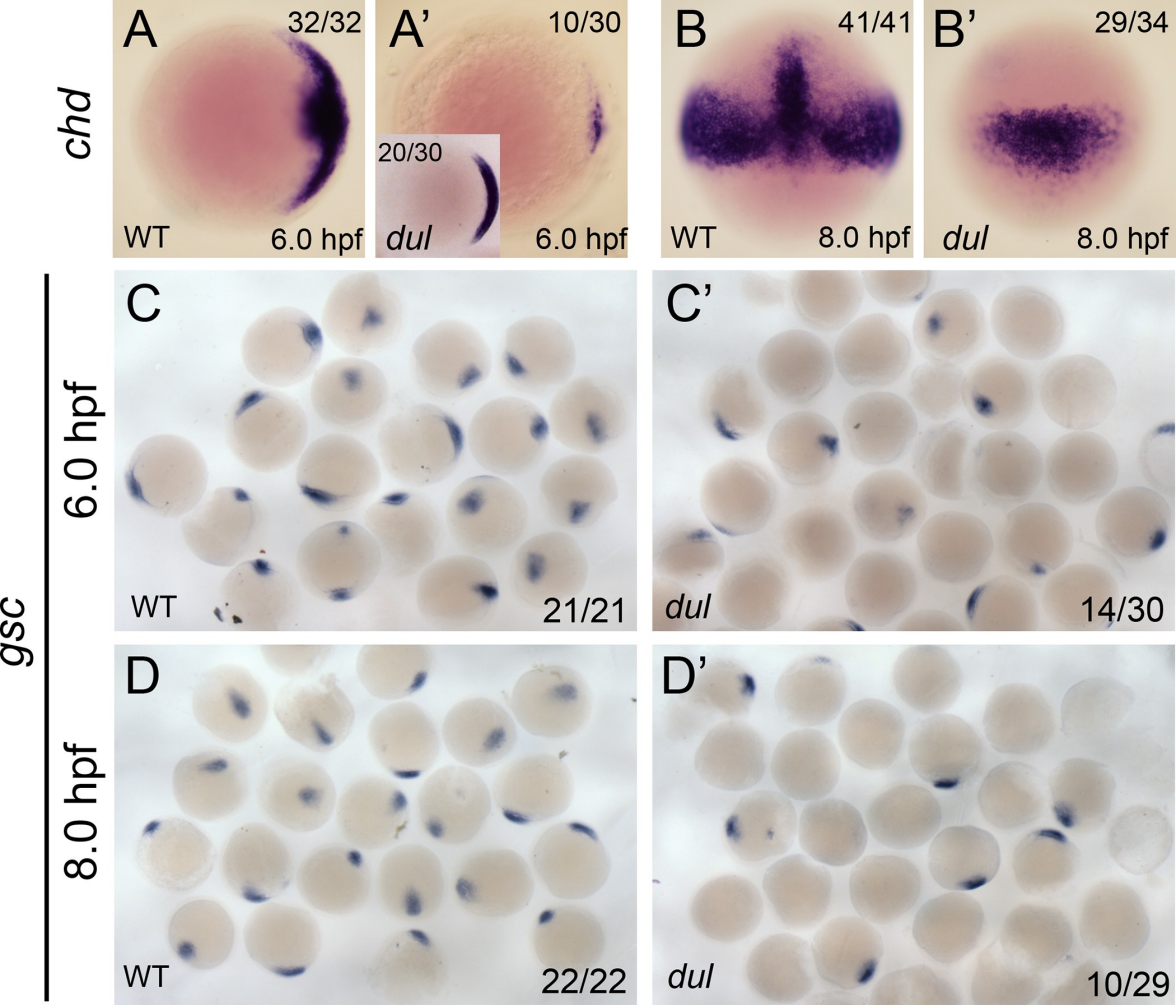
Dorsal markers are reduced in *dullahan* mutants. *chordin* mRNA expression in wild-type (**A, A’**) and *dul* (**B, B’**) embryos at 6.0 and 8.0 hpf, respectively. A and A’ are animal views, dorsal to the right. B and B’ are dorsal views. The number of embryos with the shown expression pattern of the total embryos examined is indicated in the upper right or left (inset) corner. *goosecoid* mRNA expression in wild-type (**C, C’**) and *dul* (**D, D’**) embryos at 6.0 and 8.0 hpf, respectively. The numbers in the lower right corner indicate the number of embryos with any positive *goosecoid* signal of the total embryos examined.

To investigate the ventralization of *dul* embryos, we examined the expression of two markers of dorsal tissue specification. The BMP antagonist gene *chordin* (*chd*) is expressed in the dorsal gastrula and inhibits BMP ventralizing activity [29, 30]. We also examined *goosecoid* (*gsc*) expression, a marker of the embryonic shield (dorsal organizer) [31]. In early and mid-gastrula stage embryos (6.0 and 8.0 hpf, respectively) *chd* expression was either absent or reduced in *dul* embryos (Fig 6A and 6B’). In a majority of *dul* embryos, *gsc* expression was completely absent or reduced (Fig 6C and 6D’). These results show that dorsal tissue specification is severely impaired in *dul* embryos. Since the margin separating the blastoderm from the yolk cell is significantly expanded in *dul* embryos, it is possible that dorsal determinants that originate in the vegetal region and activate a Wnt/β-Catenin signaling pathway to establish the dorsal organizer [21] are hampered in their ultimate transport to the dorsal region, resulting in ventralization.

### *p09ajug* is an allele of polo-like kinase-1

**Fig 7:**
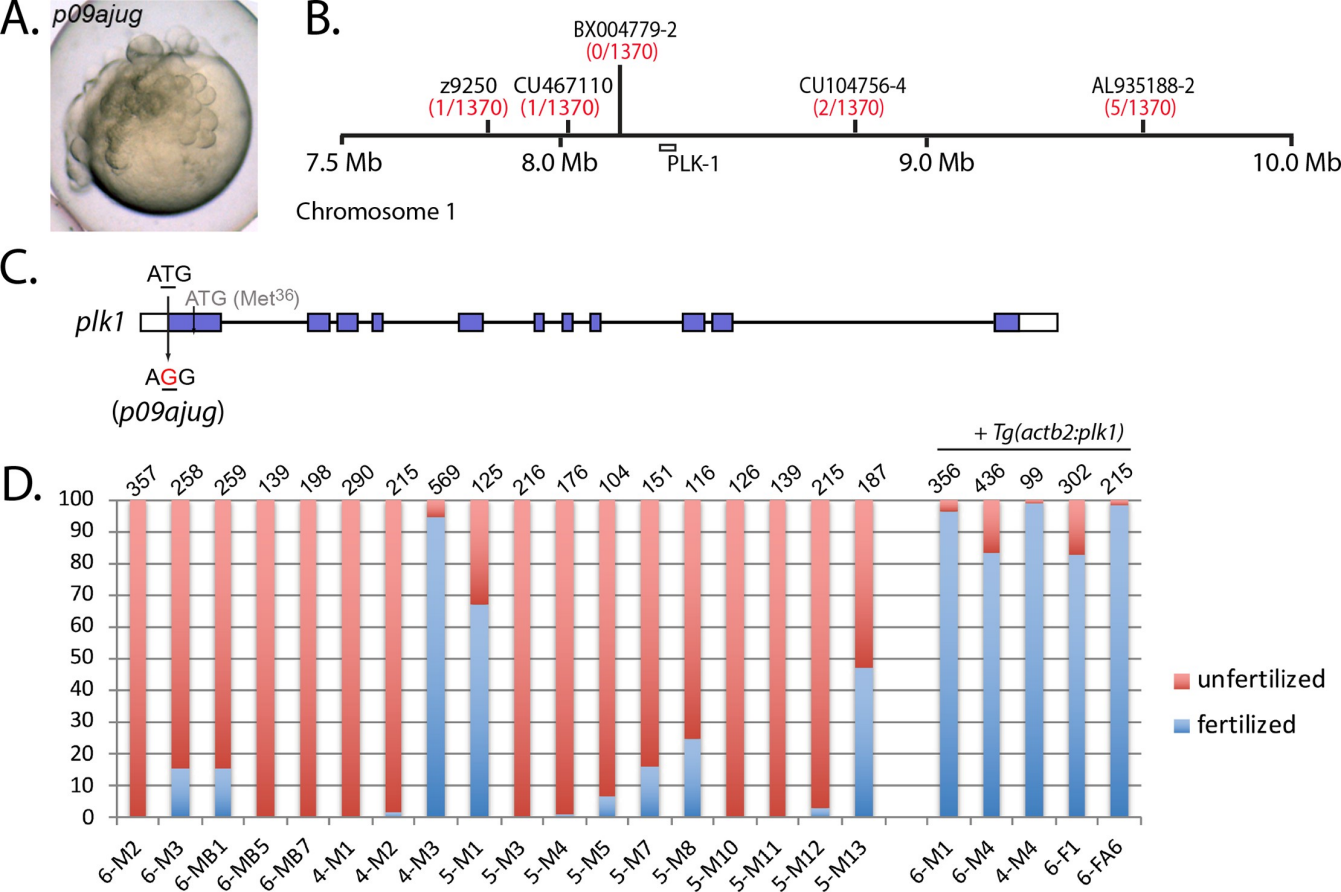
*p09ajug* is an allele of *polo-like kinase-1*. **A.** Embryos of *p09ajug* mutant females exhibit an irregular cleavage phenotype. **B.** The *p09ajug* mutation maps to chromosome 1 within a 700 kb interval flanked by SSLP markers, CU467110 and CU104756-4. **C.** Genomic structure of *plk1* indicating the T to G change in the start codon. **D.** Homozygous *p09ajug* male sterility can be rescued with *Tg(actb2*:*plk1)*. Each bar represents a different fish, where M = male and F = female. The first number in the fish name (6-, 4-, 5-) represents a particular fish family, with individual fish identifying information following that number. Total number of embryos scored is indicated at the top of each bar.

In our maternal-effect mutant screen, we identified an additional irregular cleavage mutant that resembled *mixed up* and *disarray*, called *p09ajug* (Fig 7A). Within this mutant line, we also identified a highly penetrant male sterile phenotype (Fig 7D). We mapped the *p09ajug* male sterile mutation using bulk segregation analysis to chromosome 1 between SSLP markers z11369 and z7573. The maternal-effect mutation mapped to the same location, suggesting that both phenotypes were caused by the same mutation. However, after several generations we no longer identified homozygous *p09ajug* mutant females and all homozygotes were males, suggesting a role in female sexual differentiation. Further fine mapping narrowed *p09ajug* to a 700 kb interval on chromosome 1 (1370 meiotic events; Fig 7B). No genetic recombinants were found at SSLP BX004779-2, which is 114 kb upstream of the *polo-like kinase 1* (*plk1*) gene (Fig 7B).

Sequencing of *plk1* cDNA prepared from *p09ajug* ovary tissue revealed a missense mutation, T to G, in the start codon ATG to AGG (Fig 7C). Loss of the initiation methionine is expected to result in translation initiating at the next downstream ATG (Met^36^) within the same exon leading to an amino-truncated Plk1^p09ajug^ protein. Zygotic null alleles of *plk1* display early embryonic lethality [32, 33]. Thus we postulate that the *plk1*^*p09ajug*^ allele is a hypomorphic mutation that provides sufficient zygotic activity but insufficient function for male fertility and maternal regulation of the cleavage stage. Interestingly, alignment of the N-terminus of vertebrate PLK1 homologs indicates relatively low conservation compared to most other regions of the protein (S2 Fig).

To further investigate if *p09ajug* corresponds to *plk1*, we generated a transgene with the *beta-actin2* promoter driving expression of *plk1* (*Tg(actb2*:*plk1)*) in the *p09ajug* mutant background. To facilitate the identification of carriers, the transgene vector also expresses GFP under a cardiac promoter, generating embryos with a green heart. Homozygous *p09ajug* mutant males and females containing the *plk1* transgene were fertile (Fig 7D) and females produced all normal progeny, indicating that *Tg(actb2*:*plk1)* can rescue the *p09ajug* male sterile, maternal-effect and female development phenotypes. Altogether these experiments demonstrate that *p09ajug* is a hypomorphic allele of *plk1*.

Plk1 functions in the maturation of the centrosome and is important in promoting the G2 to M cell cycle transition through the activation of Cdc25A phosphatase (reviewed in [34]). Interestingly, a study using BI 2536 chemical inhibition of Plk1 demonstrated that Plk1 is required for the first mitotic division in the mouse embryo [35]. In addition, loss of *plk1* function in *C*. *elegans* revealed a defect in uniting the maternal and paternal genomes during the process of fertilization, resulting in a “paired nuclei” phenotype [36]. Surprisingly, this phenotype persisted in the subsequent mitotic divisions during early embryogenesis [36]. More recent studies have demonstrated that wild-type Plk1 is required for the formation of a novel three-way membrane junction that promotes pronuclear fusion in *C*. *elegans* [37]. Interestingly, this structure does not normally form during subsequent somatic division in wild-type embryos, explaining the persistence of dual nuclei in temperature-sensitive *plk1* mutants [37]. In the future it will be interesting to examine the possible role of *plk1* during parental genome union at fertilization in zebrafish. However, an analogous role will likely be mechanistically distinct, since karyomere membrane fusion is involved during early zebrafish development, which requires maternal Bmb protein [17]. Moreover, a corresponding Bmb homolog appears to be lacking in the *C*. *elegans* genome [37, 38].

### Screeching halt encodes stem loop binding protein 2

**Fig 8:**
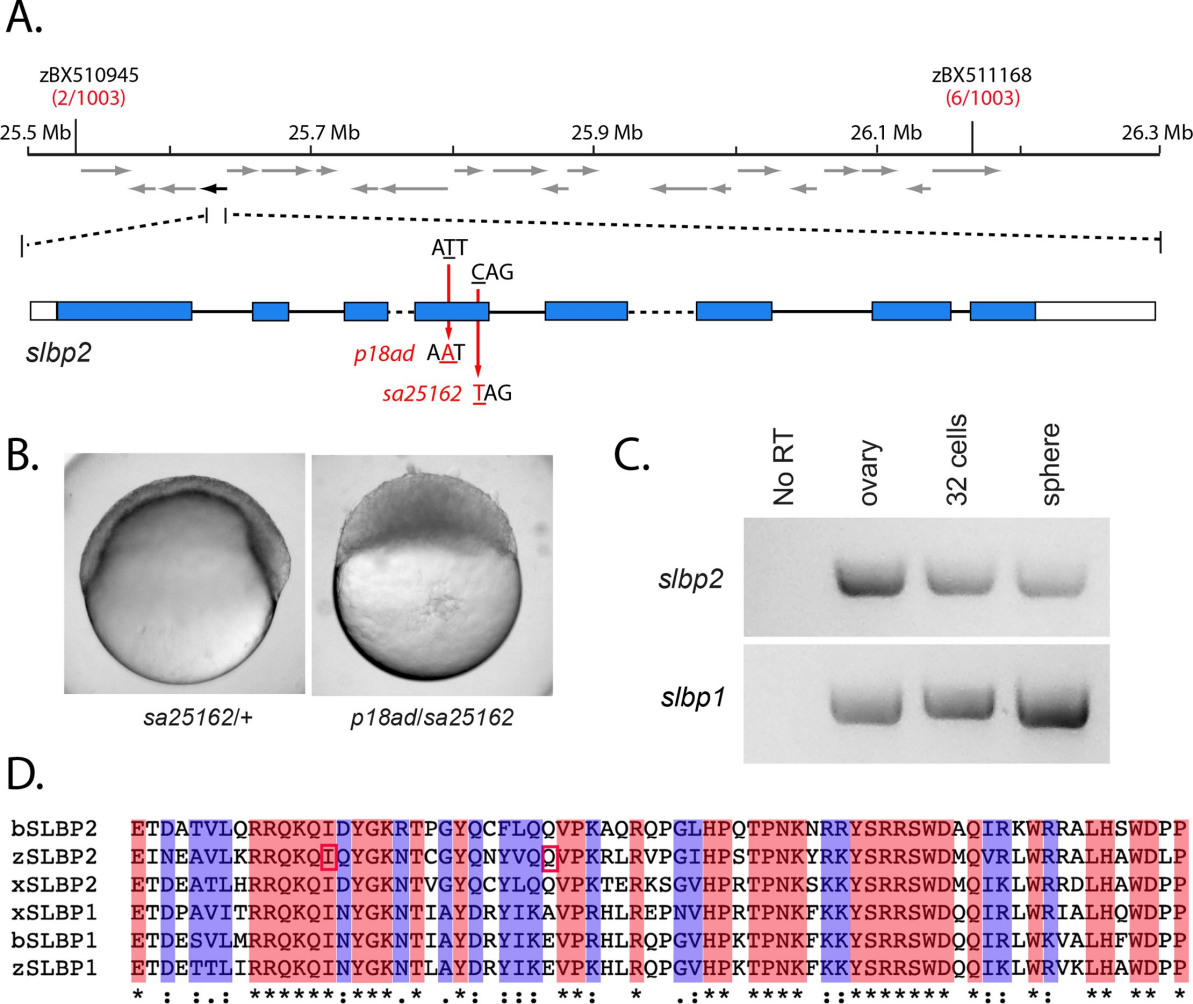
*screeching halt* encodes SLBP2. **A.** The *srh* mutation maps to a 600 kb interval on chromosome 21 flanked by zBX510945 and zBX511168. Recombinants identified between the mutation and the marker per total meiotic events examined is noted below each marker in red. The interval contains 21 predicted ORFs (arrows). The black arrow (on the reverse strand) corresponds to *slbp2*. The predicted exon-intron structure is indicated below. Note: intron 3 (568 bp) and intron 5 (1961bp) are not drawn to scale (dashed lines) due to size. Mutations corresponding to *srh*^*p18ad*^ (T to A, Iln to Asn) and *srh*^*sa12562*^ (C to T, Glu to stop) map to exon 4. **B.** The *sa25162* allele fails to complement *srh*^*p18ad*^. Embryos from *sa25162*/+ females and ten *p18ad/sa25162* trans-heterozygous females, with at least 50 embryos per female (n = 880), shown at 6 hpf. **C.** RT-PCR of *slbp2* (top) and *slbp1* (bottom) from wild-type cDNA (ovary, 32-cell and sphere stage). **D.** SLBP RBDs from zebrafish (zSLBP1 and zSLBP2), Xenopus (xSLBP1 and xSLBP2) and Bovine (bSLBP1 and bSLBP2) were aligned using Clustal Omega [53]. The Iln residue that is mutated to Asn in *srh*^*p18ad*^ and the Glu residue that is mutated to a stop codon in *slbp2*^*sa12562*^ are boxed in red. The ‘*’ indicates identical residues, ‘:’ or ‘.’ indicate similar residues.

We next investigated the molecular nature of the *screeching halt* (*srh*^*p18ad*^) mutant gene, which causes a similar mid-blastula arrest phenotype to the *mcm3l* maternal-effect mutation, *p10umal*. To determine the molecular nature of the *srh* defect, we positionally cloned the corresponding mutant gene. We examined 1003 meioses and mapped *srh* to a 600 kb interval on chromosome 21 (Fig 8A). The meiotic recombination frequency of the flanking markers predicts that the mutation resides in the third of the genetic interval proximal to zBX510945 (Fig 8A). Sequence analysis of genes in this region revealed a T to A base change in the RNA binding domain encoded in the *stem loop binding protein 2* (*slbp2*) gene, resulting in the change of a conserved Isoleucine to Asparagine (Fig 8A and 8D).

Since the *srh* mutation encodes an Ile to Asp missense change, we sought to verify that this lesion indeed disrupted SLBP2 function. A search of the ZMP collection revealed *slbp2* alleles containing premature stop codons [27]. One such allele, *sa25162*, contains a C to T base-pair change at nucleotide 469 of the ORF, converting Gln^157^ to a stop codon (Fig 8A and 8C). Trans-heterozygous females were generated and their progeny examined. As expected, the *slbp2*^*sa25162*^ allele failed to complement *srh*^*p18ad*^, and 100% of the mutant embryos exhibited the mid-blastula developmental arrest phenotype (Fig 8B; n = 880 from 10 trans-heterozygous females). This demonstrates that the *srh* phenotype is due to a defect in Slbp2 function.

The related SLBP1 protein functions in processing and stabilizing core histone transcripts and promoting translation of histone proteins, the production of which is tightly regulated with cell cycle progression and DNA replication (reviewed in [39]). SLBP1 associates with a specialized stem loop structure located in the 3’ UTR of core histone mRNAs. Its role is analogous to that of Poly-A binding protein, which stabilizes and promotes translation of poly-A containing mRNA transcripts. *Slbp2* mRNA is expressed mainly in the ovary of frogs [40] and several mammals [41]. It has been speculated that SLBP2 in bovine can stockpile a significant supply of histone mRNA in the oocyte for the ensuing cleavages of the fertilized egg [42]. Since the translation activation and RNA processing domains are not conserved in SLBP2 (S3 Fig), this may be achieved by SLBP2 stabilizing and preventing bulk histone transcripts from being translated during oogenesis. It is postulated in Xenopus that subsequently, during egg activation, SLBP2 is degraded, allowing SLBP1 to associate with the 3’ UTR stem loop to promote mRNA processing and core histone translation [40].

We examined the mRNA expression of *slbp2* and *slbp1* in the ovary and during early zebrafish development. RT-PCR revealed expression of *slbp2* in the ovary, during the cleavage stage, and at 4 hpf (sphere stage), a post-MBT stage (Fig 8C). Comparably, *slbp1* expression was detected at all corresponding stages (Fig 8C), consistent with previous data that also showed *slbp1*, but not *slbp2* expression at later embryonic and larval stages [43, 44]. Loss of zygotic Slbp1 function in the zebrafish leads to normal early embryonic development, but causes retinal developmental defects by 2 dpf, in addition to other morphological defects by 3 dpf [44]. Thus, although *slbp2* and *slbp1* are co-expressed in the ovary and during the early cleavage stage, they exert distinct developmental defects.

**Fig 9:**
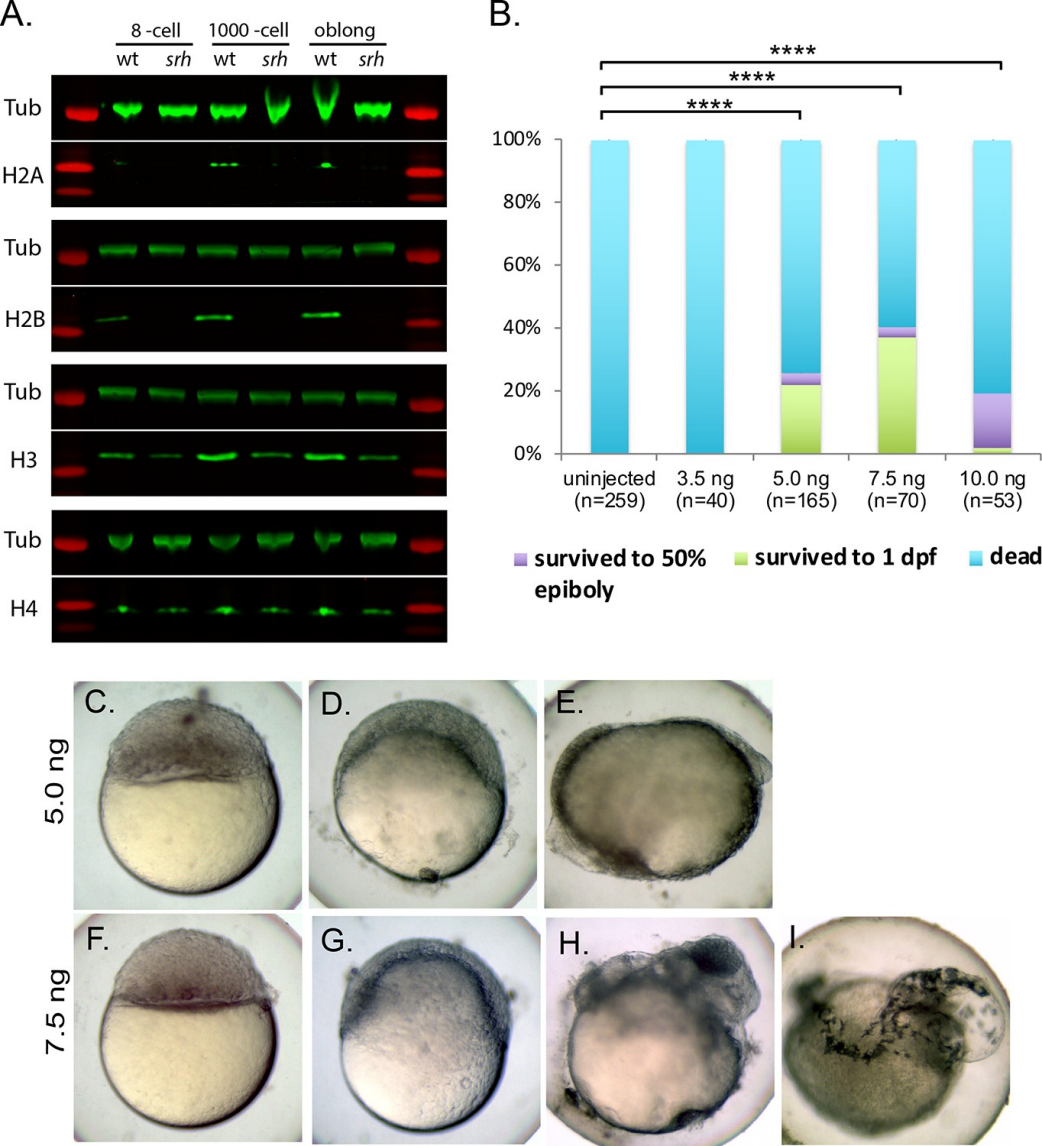
Slbp2 is required for histone production during early development. **A.** Western blot analysis of the four core histones in wild-type and *srh* embryos. Anti-α-tubulin was used as a loading control. **B.** The *srh* developmental arrest phenotype can be rescued by injecting total histone protein into one-cell stage *srh* embryos. P-values were determined using a Student’s t-test. ****p< 0.0001. *srh* embryos injected with 5 ng (**C**-**E**) or 7.5 ng (**F**-**I**) of whole histone. **C** and **F**, lateral views imaged at 5 hpf. **D** and **G** lateral views imaged at 6 hpf. **E** and **H** were imaged at 24 hpf. **I** was imaged at 48 hpf. Note the head formation and eye pigmentation in **H** and the presence of melanocytes in **I**.

Next, we investigated whether disrupted Slbp2 function affects maternally-supplied histone protein levels during early development. Protein extracts were prepared from embryos obtained at the 8-cell to 3.5 hpf stages from wild-type and *srh*^*p18ad*^ females and used for western blotting. Antibodies that specifically recognize each of the core histones revealed that H2A and H2B were almost completely absent in *srh* embryonic extracts compared with wild-type extracts at each stage tested, while H3 and H4 were notably reduced (Fig 9A; S4C and S4D Fig). We also examined histone levels in unfertilized eggs. Like embryonic extracts, H2B levels were significantly reduced in unfertilized eggs, however, H3 and H4 were not significantly reduced (S4A and S4B Fig). These results indicate that Slbp2 functions in producing the core histone protein levels that act during the maternally-controlled cleavage stages. Since Slbp1 is expressed during both maternal and zygotic periods (Fig 8C), the low levels of core histones remaining in *srh*-derived extracts may be products of Slbp1 activity. Furthermore, the anti-H3 antibody may also recognize histone variants, such as H3.3, which are polyadenylated and do not require SLBP activity for expression (i.e., do not have a 3’ UTR stem loop). This may also contribute to the low levels of H3 still detected in *srh* mutants.

To determine if the reduction in histone proteins causes the *srh* developmental arrest phenotype, we injected into one-cell stage *srh* embryos whole histones derived from calf thymus, which contain all four core histones. Uninjected or *srh* embryos injected with 3.5 ng of whole histones never initiated epiboly (Fig 9B). In contrast, a substantial fraction of embryos injected with 5 or 7.5 ng of whole histones, initiated epiboly and survived to 1 dpf. Remarkably, a few survived to 2 dpf (Fig 9B–9I). These data indicate that *srh* mutants are deficient in histone protein production, which leads to an arrest in their development at a mid-blastula stage.

Interestingly, knockdown of SLBP in dsRNA transgenic mouse oocytes also leads to an early developmental arrest, and the phenotype is rescued by injecting histone protein [45]. Recently, He et al., employed a reverse genetics approach to examine the function of zebrafish SLBP2 by generating indel alleles using CRISPR-Cas9 [43]. Interestingly, they demonstrated partial rescue of a chromatin phenotype in the arrested embryos (also reported in *srh* mutants, [13]) by introducing an H2B transgene into the mutant background. However, replenishing H2B failed to rescue the developmental arrest phenotype, indicating that at least one other core histone is necessary, which indeed was supplied in our rescue experiments.

Like SLBP1, the RNA binding domain (RBD) of zebrafish SLBP2 is highly conserved to the frog and mammalian homologs (Fig 8D). However, outside of the RBD, the sequence is far less conserved (S3 Fig). Furthermore, in zebrafish, bovine and frog SLBP2, the regions important for translation activation [46] and RNA processing [47] are not conserved (S3 Fig). Thus, it is possible that SLBP2 serves to maintain and stabilize maternal histone transcripts and prevent core histone mRNAs from being translated, as previously proposed [40, 41]. Indeed, He et al. showed a strong reduction in core histone protein transcripts, *h2a*, *h2b*, *h3*, and *h4*, in the ovary and cleavage stage of zebrafish *slbp2* maternal mutants [43]. Surprisingly, however, we found little to no reduction in H3 and H4 protein in unfertilized eggs, although clear reductions were evident at later cleavage stages (Fig 9A and S4 Fig). Since SLBP2 protein is present throughout oogenesis and during the cleavage stage, it may be displaced by SLBP1 to allow for core histone production of the maternal histone mRNA or function in conjunction with other proteins to mediate their translation. Our data are consistent with this model, since the absence of maternal SLBP2 would lead to decreased stability of histone mRNA and consequently reduced levels of maternal core histones. This regulatory mechanism is essential to maintain the optimal level of core histones during cleavage, since a histone excess or deficit can disrupt normal progression of the MBT (see below).

The possibility of SLBP2 having a more direct role in histone production should not be completely excluded. Transgenic dsRNA knockdown experiments in mice have shown that functional SLBP is required for histone H3 and H4, but not H2A or H2B accumulation in the mouse oocyte [45]. Mice have only one functional copy of *Slbp*, which is most homologous to *Slbp1*, and *Slbp2* is instead a pseudogene in mice [41]. Our data indicates that Slbp2 is required for H2A and H2B accumulation and to a lesser degree H3 and H4 accumulation (Fig 9A and S4 Fig). This combined with the presence of *slbp1* mRNA in the oocyte (Fig 8C), raises the possibility that zebrafish Slbp1 may augment H3 and H4 production, as is the case for SLBP in the mouse. It should be noted that zebrafish Slbp2 protein (326 residues) is much larger than the bSLBP2 (152 residues) or xSlbp2 (250 residues) counterparts (S3 Fig). Whether any translation activation and/or RNA processing-associated activity resides in these undefined regions of zebrafish Slbp2 remains to be determined.

Recently, it has been demonstrated that decreasing or increasing endogenous levels of maternal histones can lead to premature or delayed ZGA, respectively [48, 49]. Indeed, three of seven tested zygotic genes were not expressed in *srh* mutants [13]. Future experiments are needed to address the extent that ZGA is affected in *srh* mutant embryos and to what degree maternal histone concentration plays a role in promoting the MBT.

### Mid-blastula arrest mutants

Here, we identified the molecular nature of two maternal-effect mutant genes that cause an arrest in development proximal to the MBT and encode maternal-specific proteins important for cell cycle progression. These two developmental arrest mutants together with a third we previously identified, *brambleberry*, all arrest at the same mid-blastula stage, yet they have very distinct cellular defects and encode diverse factors (Mcm3l, a DNA replication initiation factor; Slbp2 regulates histone protein production; Brambleberry regulates nuclear karyomere fusion [17]). There may be a developmental checkpoint at this stage that causes the arrest or alternatively each mutant may similarly fail to express key zygotic gene(s) required to initiate further development. Consistent with the latter is that a similar stage of developmental arrest is caused by inhibition of wholesale transcription by actinomycin D treatment [50]. Further studies are required to distinguish between these possibilities.

In summary, we have performed a chemically-induced mutagenesis screen and characterized 9 maternal-effect mutants with defects in different aspects of the cleavage stage of development. All of the corresponding mutations were mapped to relatively narrow genetic intervals (Table 1) and several of the mutant genes were cloned. Our molecular genetic approach has revealed critical maternal-specific factors, as well as a hypomorphic allele of a more broadly-acting factor (*plk1*), that function in the cleavage stage of development. Our study also provides novel maternal-effect mutants that are expected to aid in elucidating the molecular mechanisms regulating cell division of the unusually large blastomeres during the checkpoint-free cleavage stage of vertebrate development.

## Materials and methods

### Ethics statement

This research was approved by the University of Pennsylvania Institutional Animal Care and Use Committee (IACUC).

### Fish stocks

The following mutant stocks were generated through a large-scale ENU mutagenesis screen: *p04anua*, *p01aiue*, *p40atuz*, *p09ajug*, *p15uzat*, *p86batl*, *p09batl*, *p10umal*, *p22atuz*. *srh*^*p18ad*^ was reported in [13]. Zebrafish genome assembly version 9 (Zv9) was used for linkage analysis to determine the chromosomal map position of the corresponding mutant alleles. All wild-type samples used in experiments were Tupfel long fin (TL) or corresponding siblings of mutant alleles. The *mcm3l*^*sa1624*^ and *slbp2*^*sa25162*^ mutant allele strains were obtained from the Zebrafish Mutation Project [27]. The *p22atuz*, *p18ad*, *p10umal*, *p09ajug* and *mcm3l*^*sa1624*^ alleles were genotyped using KBiosciences Competitive Allele-Specific PCR genotyping system (KASP, KBiosciences). The following sequences were sent to KBiosciences to generate corresponding assay mixes: *p22atuz*: 5’- CAATGGTTTACATGCGTTTATCAACTCTGCTAATACTGTCATGTG CTNNT(A/T)GAGAATGTTAGCGGGCAGCTGAAGGATCAGACCGCAGAAGTGCAGGAAG;

*p18ad*: 5’- TCTCATTTTGAGATCAATGAGGCTGTTTTGAAGCGTAGGCAAAAGCAG A(A/T)TCAGTATGGGAAGAATACCTGTGGCTACCAGAACTACGTTCAGCAGGTTC-3’;

*p10umal*: ATTTTAAAATATTGTAATGCACATGCTGATGCGCGTGTAGGAGAA[A/C]TGG ATACTGGGTTAGAGGACCTCGAGCTGAGAGAGTCACAGAGGGAATATC; *p09ajug*: 5’-GGACGTTAGGGTGTATTTTTGTACTTAAGAGCATTTGTAGTGTACAACGA[T/G]GAGTGCTGCAATTGCAAAGCCATCGGCGAAGCCATCGGCTCACGTCGAT-3’. *mcm3l*^*sa1624*^ and *slbp2*^*sa25162*^ sequences were obtained from the Sanger Centre (sanger.ac.uk). The *plk1*^*p09ajug*^ allele was also genotyped using derived Cleaved Amplified Polymorphic Sequences (dCAPS). The following primer sets were used to a generate a 162 bp amplicon: dCAP-plk1-F1: 5’-TAAGAGCATTTGTAGTGTAC**AtCGA**-3’; dCAP-plk1-R1: 5’-CCCAAAAAGCGACC TCTCATGTATC-3’. The bold nucleotides correspond to a partial Cla-1 restriction site that is generated in wild-type genomic DNA (ATCGAT) and disrupted in a mutant DNA (ATCGA**G**). The lower case t was engineered into the primer to create the partial Cla1 site. The resulting 162 bp amplicon was digested with ClaI to produce 142 and 20bp fragments in wild-type DNA, which were resolved and visualized on a 2% LE agarose gel.

### Whole mount antibody staining and in situ hybridization

For fertilization time courses, embryos were collected at 10 mpf (controlled matings) and fixed at 3-minute intervals in 4% paraformaldehyde/PBS (PFA) overnight. Immunofluorescence, DAPI staining and confocal imaging were performed as previously described [17] using the following antibodies: PCNA (1:500, Abcam; Ab-29), phospho-histone H3 (1:200, Millipore Sigma; 06–570). Whole mount *in situ* hybridization was performed as previously reported [20].

### Chromosomal mapping of maternal-effect mutations and positional cloning of *p10umal* and *srh*

Mutants were mapped using bulk segregation analysis [24]. The closest linked SSLP to each mutation is listed in Table 1. SSLPs designed for fine mapping of *p10umal*, *p09ajug* and *srh*^*p18ad*^ are listed in S3 Table. Complete ORFs of *mcm3l* and *slbp2* were amplified by RT-PCR using the following primer sets: *mcm3l*-ORF (for)-5’-GCTTGGTTTGGTTGCTTCAT-3’, *mcm3l*-ORF (rev)-5’- ATGAGAAACACCACATCCT CTG-3’ and *slbp2*-ORF (for)-5’-GCCAAAATCATG ACAACACG-3’ and *slbp2*-ORF (rev)-5’- TCAAAATCTCGAAGGCTGCT-3’. PCR products were sequenced on both strands by the University of Pennsylvania DNA Sequencing Facility.

### Transgene, Tg(*actb2:plk1*), generation

The *pkl1* ORF was amplified from testis cDNA using the following primer pairs: plk1-pentr-F1- 5’-CACCATGAGTGCTGCAATTGCAAAGCC-3’ and plk1-pentr-R1-5’- TTAGCGTGCTGAAGTAGCA GCTGTTGTGC-3’ and subsequently cloned into pENTR-TOPO (Invitrogen) to generate a middle entry clone, which was subsequently cloned into pDestTol2CG2 (contains a cardiac myosin light-chain GFP cassette) [51] along with a 5’ entry clone containing the *beta-actin2* promoter [52] and a 3’ polyA entry clone using Gateway cloning technology (Invitrogen). 25pg of pDestTol2CG2B-actin-*plk1* DNA along with 25 pg of Tol2 Transposase mRNA [51] was injected into the cytoplasm of one-cell stage embryos obtained from a *p09ajug* heterozygous female crossed to a *09ajug* heterozygous male. Founder fish were identified by screening their corresponding progeny for cardiac GFP. A *p09ajug* (-/+) founder fish was subsequently in-crossed to *p09aug* to produce F1 *p09ajug* homozygous and heterozygous fish carrying the transgene, *Tg(actb2*:*plk1)*. The transgene segregates in a Mendelian fashion through multiple generations, i.e. in an outcross, 50% of the progeny inherit the transgene, as indicated by green fluorescence in the heart and by genotyping for the transgene, indicating that it is a single transgene insertion.

### RT-PCR

RNA was prepared from ovaries or embryos of the indicated stages using Trizol reagent (Invitrogen) according to manufacturer's instructions. cDNA was generated using Superscript II reverse transcriptase (Invitrogen) according to manufacturer's instructions. The following primer pairs were used to detect *mcm3* transcripts: *mcm3* (for)-5’- GGAAGAGGAGCTCCAGGTTT-3’, *mcm3* (rev)-5’- AATCAAACCCACCGACTGAG-3’. The following primers were used to detect *mcm3l*: *mcm3l* (for)-5’- AAGCTGGTGA AGCCAGTGTT-3’, *mcm3l* (rev)-5’- ATGAGAAACACC ACATCCTCTG-3’. The following primer pairs were used to detect *slbp1* and *slbp2*: *slbp1* (for)-5’-GATGAGGTGGAGGAACAGGA-3’, *slbp1* (rev)-5’- TTGATGAGCATTGGGATTCA-3’, *slbp2* (for)-5’-GCCAAAATCATGACAACACG-3’ and *slbp2* (rev)-5’ CAAGCAAGCTCTGCAGT TGA-3’.

### Western Blotting

Embryos were dechorionated and blastoderms were manually removed from the yolk using forceps and snap frozen in liquid nitrogen. Per experiment, equal numbers of embryos were analyzed for each developmental stage. For fluorescent western blots samples were boiled in SDS loading buffer at 98°C for 10 min and run on 4–12% polyacrylamide NuPAGE Bis-Tris gels (NP0321BOX; ThermoFisher Scientific). Proteins were blotted onto a nitrocellulose membrane using transfer buffer (15% methanol) at 220 mA for 75 min. 5% milk/TBS was used to block membranes for 1 hour at RT or overnight at 4°C. Primary antibodies were incubated for 1 hour at RT or overnight at 4°C. Secondary antibodies were incubated for 45 min at RT. 5% milk/TBS-Tween was used to dissolve primary and secondary antibodies. Between incubations washes were done in TBS-Tween. Before detection, membranes were first washed with TBS-Tween, then desalted in water, dipped in methanol and allowed to air dry. Membranes were analyzed on an Odyssey Infrared Imaging System (LI-COR). As a loading control, α-tubulin was examined visually on all blots. Histone antibodies were obtained from Abcam (Cambridge, MA). Anti-H2A (ab18255; 1:1,000), anti-H4 (ab10158; 1:1,000), anti-H2B (ab1790; 1:3,000) and anti-H3 (ab1791; 1:10,000) were the primary antibodies used for western blotting. Anti-α-tubulin (Sigma, T6074) was used as a loading control (1:20,000). Three biological replicates were examined.

For quantification of westerns, histone content for a given sample was normalized to tubulin and then a scaling factor was computed from the wild-type sample run on the same membrane (such that wild-type was always assigned a value of one). For each treatment we performed linear model (ANOVA) test with the scaled concentration as a function of protein, genotype and interaction between the two to identify significant interaction terms. Finally, we performed a post-hoc Tukey's range test to generate the P-values for each specific contrast reported in S4 Fig.

### Histone Microinjections

A total of 3.5 ng, 5 ng or 7.5 ng of whole histones (Sigma H9250) was injected into one-cell stage embryos derived from wild-type or *srh* mutant mothers. Embryos were examined at 5, 6, 24 and 48 hpf to assay for rescue.

## Supporting information

S1 Fig N-terminal alignment of vertebrate Mcm3 homologs. Approximately 50 residues at the N-terminus of Mcm3 homologs corresponding to *Danio rerio* (fish), *Taeniopygia guttata* (bird), *Homo sapien* (human), *Xenopus laevis* (frog) and *Gopherus evgoodei* (turtle) were aligned using the PRALINE alignment tool [54]. The predicted Met used as a start codon in the *p10umal* allele is at position 40 (black box) in this alignment. Consistency values for each amino acid position ranging from 1 to 10(*) were assigned by the PRALINE alignment tool [54].
(TIF)

S2 Fig Alignment of vertebrate PLK1 homologs. PLK1 homologs corresponding to *Danio rerio* (zebrafish), *Gallus gallus* (chicken), *Homo sapien* (human), *Xenopus laevis* (frog) and *Podarcis murali* (lizard) were aligned using the PRALINE alignment tool [54]. The predicted Met used in the *p09ajug* allele is at position 51 in this alignment. The N-terminal most residues expected to be absent from Plk^p09ajug^ (black bar) contain 38 amino acids of relatively low conservation, followed by 10 amino acids of higher conservation. Consistency values for each amino acid position ranging from 1 to 10(*) were assigned by the PRALINE alignment tool [54].
(TIF)

S3 Fig Alignment of zebrafish, *Xenopus* and bovine SLBP1 and SLBP2. Zebrafish (zSLBP1 and zSLBP2), *Xenopus* (xSLBP1 and xSLBP2) and Bovine (bSLBP1 and bSLBP2) were aligned using Clustal Omega [53]. Black line indicates the RNA binding domain (RBD). Red box indicates the region important for translation activation (TAD) and the blue box is the region important for RNA processing (RPD). The ‘*’ indicates identical residues, ‘:’ or ‘.’ indicate similar residues.
(TIF)

S4 Fig Quantification of histone levels in *srh* vs wild-type embryos. **A.** Western blot analysis of protein extracts obtained from unfertilized eggs corresponding to wild-type (left) and *srh* (right). **B.** Quantification of blots like those in panel **A** with histone concentration normalized to tubulin controls and with WT scaled to one. N ≥ 3 replicates for all comparisons. **C.** Western blot analysis of protein extracts obtained from 2–2.5 hpf embryos corresponding to wild-type (left) and *srh* (right). **D.** Quantification of blots like those in panel **C,** with histone concentration normalized to tubulin controls and with WT scaled to one. N ≥ 3 replicates for all comparisons. Error bars represent standard deviation. P-Values calculated using Tukey's range test. Single asterisk (*) denotes p ≤ 0.01, double asterisks denotes p ≤ 0.005.
(TIF)

S1 Table Maternal-effect arrest mutants do not exhibit an obvious zygotic phenotype. (DOCX)

S2 Table Complementation crosses of *p10umal*/+ X *sa1624/sa1624*. (DOCX)

S3 Table Sequence of primer sets used in fine mapping. (DOCX)
