# Supplementary figures and images for: Molecular genetics of maternally-controlled cell divisions
Source: PLoS Genet. 2020 Apr 8;16(4):e1008652. doi: 10.1371/journal.pgen.1008652 (PMC7179931; doi:10.1371/journal.pgen.1008652)

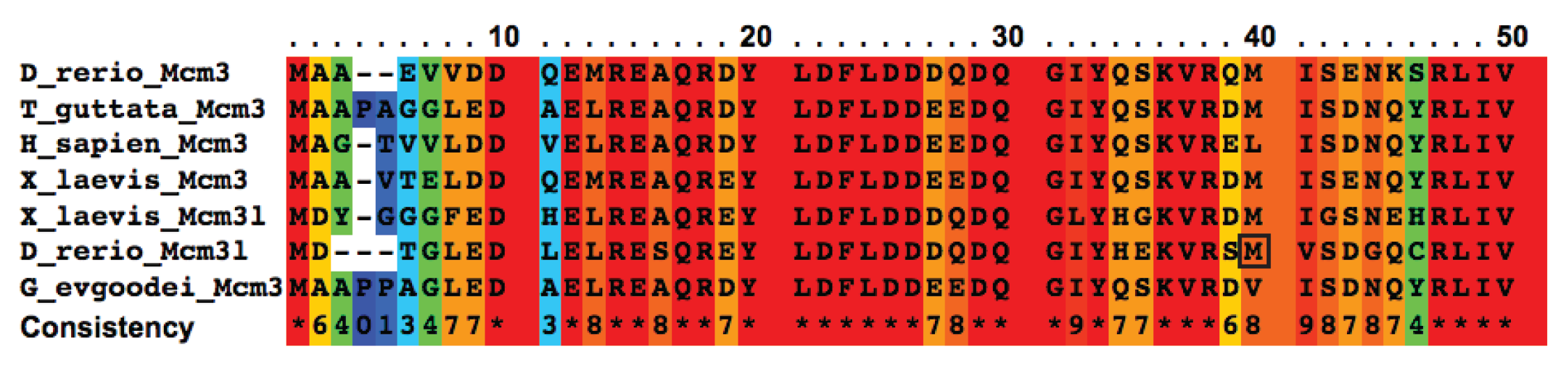

Supplement: S1 Fig — Approximately 50 residues at the N-terminus of Mcm3 homologs corresponding to Danio rerio (fish), Taeniopygia guttata (bird), Homo sapien (human), Xenopus laevis (frog) and Gopherus evgoodei (turtle) were aligned using the PRALINE alignment tool [54]. The predicted Met used as a start codon in the p10umal allele is at position 40 (black box) in this alignment. Consistency values for each amino acid position ranging from 1 to 10(*) were assigned by the PRALINE alignment tool [54]. (TIF) [file pgen.1008652.s001.tif]

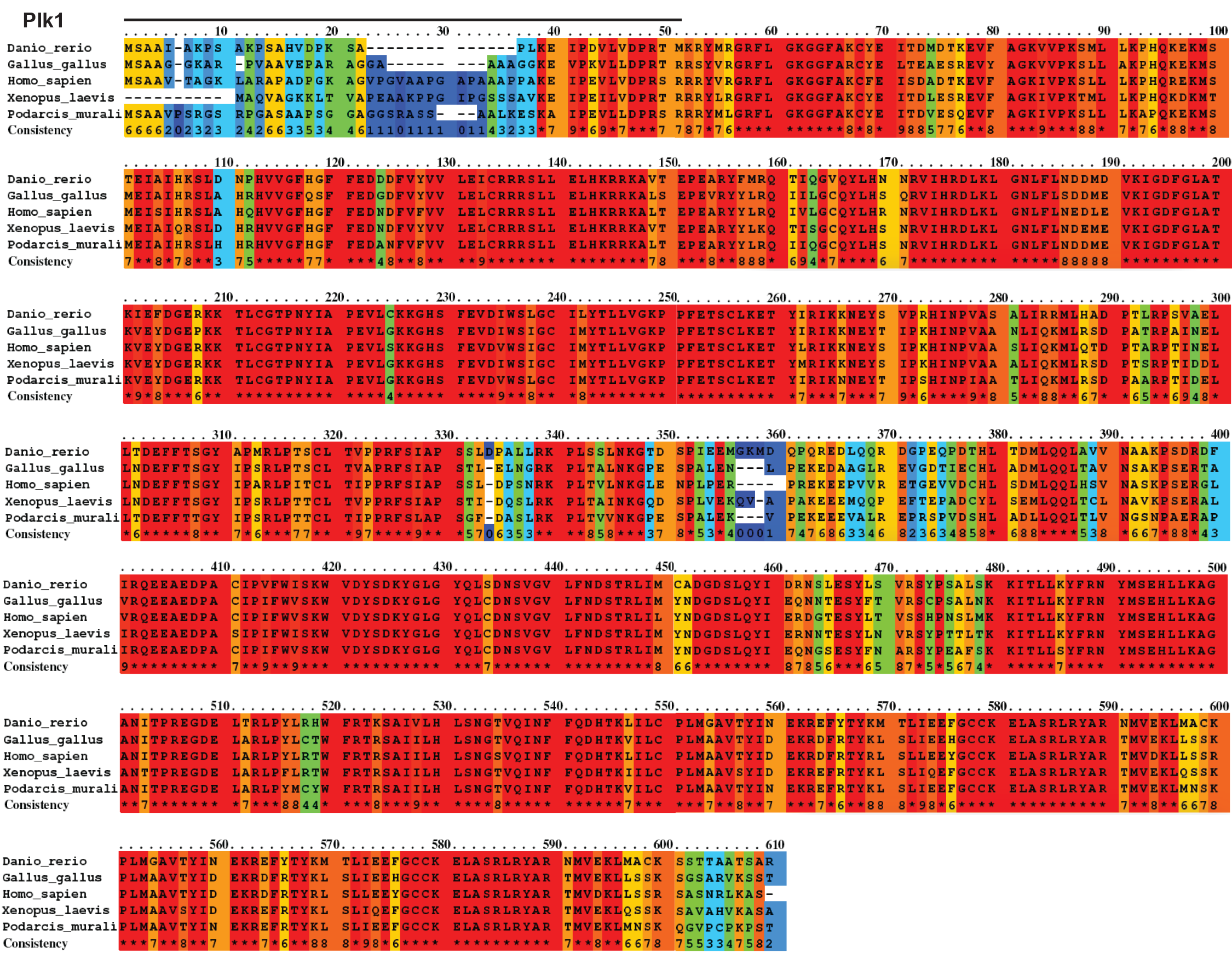

Supplement: S2 Fig — PLK1 homologs corresponding to Danio rerio (zebrafish), Gallus gallus (chicken), Homo sapien (human), Xenopus laevis (frog) and Podarcis murali (lizard) were aligned using the PRALINE alignment tool [54]. The predicted Met used in the p09ajug allele is at position 51 in this alignment. The N-terminal most residues expected to be absent from Plkp09ajug (black bar) contain 38 amino acids of relatively low conservation, followed by 10 amino acids of higher conservation. Consistency values for each amino acid position ranging from 1 to 10(*) were assigned by the PRALINE alignment tool [54]. (TIF) [file pgen.1008652.s002.tif]

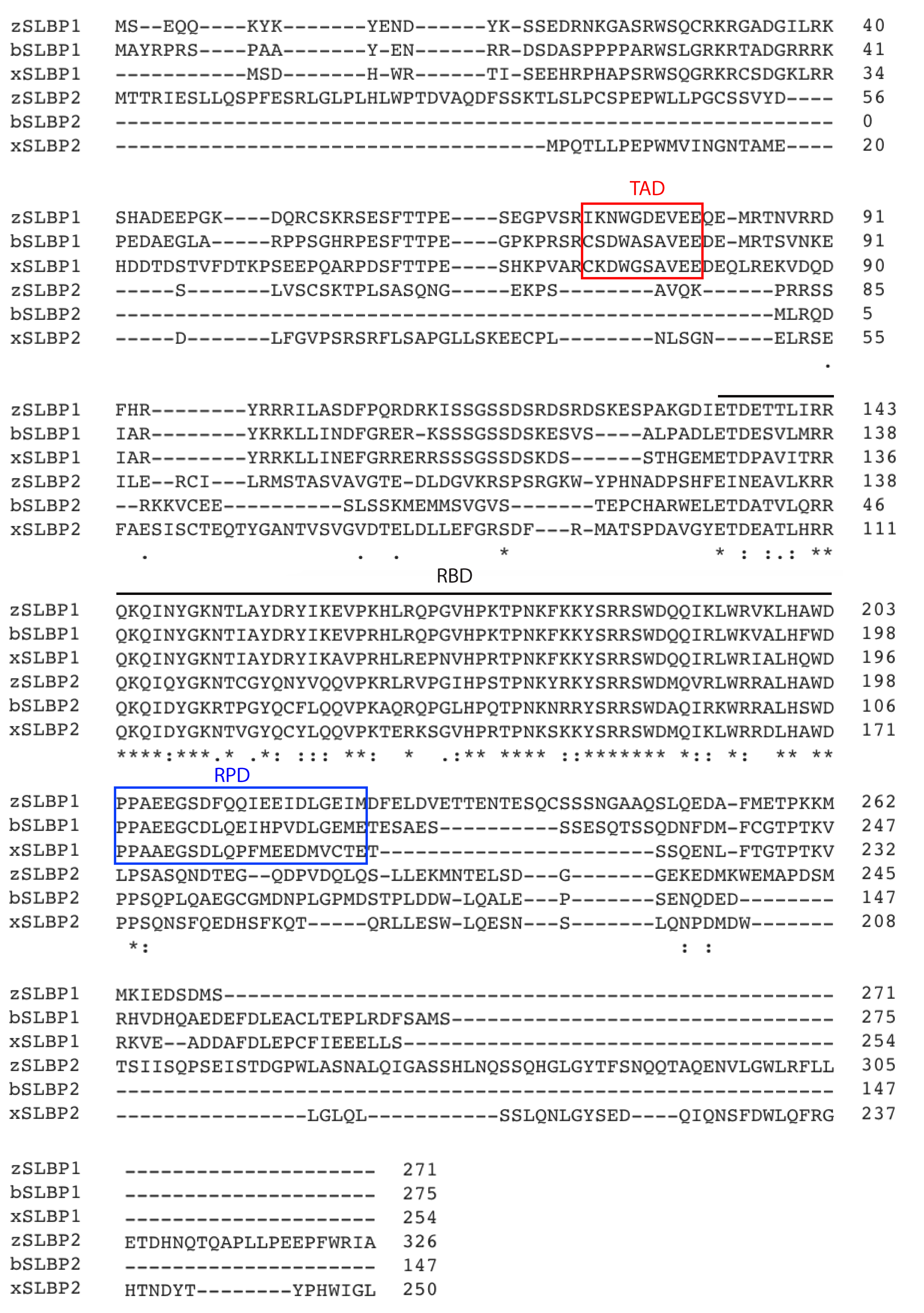

Supplement: S3 Fig — Zebrafish (zSLBP1 and zSLBP2), Xenopus (xSLBP1 and xSLBP2) and Bovine (bSLBP1 and bSLBP2) were aligned using Clustal Omega [53]. Black line indicates the RNA binding domain (RBD). Red box indicates the region important for translation activation (TAD) and the blue box is the region important for RNA processing (RPD). The ‘*’ indicates identical residues, ‘:’ or ‘.’ indicate similar residues. (TIF) [file pgen.1008652.s003.tif]

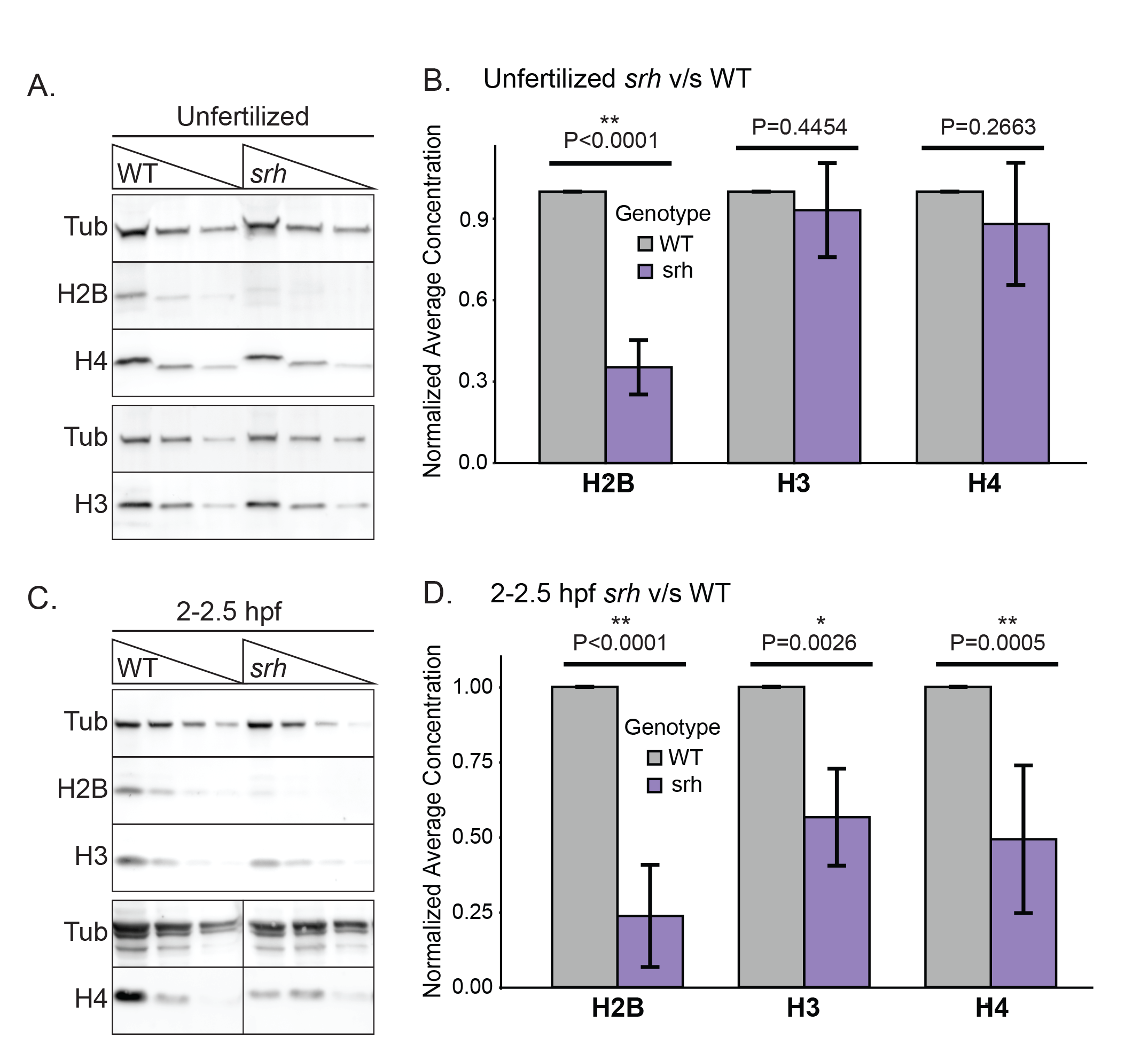

Supplement: S4 Fig — A. Western blot analysis of protein extracts obtained from unfertilized eggs corresponding to wild-type (left) and srh (right). B. Quantification of blots like those in panel A with histone concentration normalized to tubulin controls and with WT scaled to one. N ≥ 3 replicates for all comparisons. C. Western blot analysis of protein extracts obtained from 2–2.5 hpf embryos corresponding to wild-type (left) and srh (right). D. Quantification of blots like those in panel C, with histone concentration normalized to tubulin controls and with WT scaled to one. N ≥ 3 replicates for all comparisons. Error bars represent standard deviation. P-Values calculated using Tukey's range test. Single asterisk (*) denotes p ≤ 0.01, double asterisks denotes p ≤ 0.005. (TIF) [file pgen.1008652.s004.tif]
