# Supplementary material for: Molecular genetics of maternally-controlled cell divisions
Source: PLoS Genet. 2020 Apr 8;16(4):e1008652. doi: 10.1371/journal.pgen.1008652 (PMC7179931; doi:10.1371/journal.pgen.1008652)
Supplement: S1 Table — (DOCX) [file pgen.1008652.s005.docx]

**S1 Table.**

**Maternal-effect arrest mutants exhibit no obvious zygotic phenotype.**

| Cross **^a^** | Female**^b^** | Shield**^c^** | 24 hpf**^c^** |
| --- | --- | --- | --- |
|  |  |  |  |
| *bmb^p22atuz/+^* X *bmb^p22atuz-/-^* | 1 | 130/137**^d^** | 130/130 |
|  | 2 | 95/97**^d^** | 95/95 |
|  | 3 | 241/241**^d^** | 241/241 |
|  | 4 | 198/198 | 198/198 |
| *srh^p18ad^*^/^*^+^* X *srh^p18ad-^*^/^*^-^* | 1 | 298/307**^d^** | 298/298 |
|  | 2 | 219/219 | 219/219 |
|  | 3 | 90/90 | 90/90 |
| *p10umal*/*+* X *p10umal-*/*-* | 1 | 79/79 | 79/79 |
|  | 2 | 280/286**^d^** | 280/280 |
|  | 3 | 230/233**^d^** | 230/233 |
| TL X TL | 1 | 177/185**^d^** | 177/177 |
|  | 2 | 125/125 | 125/125 |
|  | 3 | 142/144 | 140/142 |

(a) Heterozygous mutant females crossed to homozygous mutant males. Tupfel Long fin

(TL) were used as a wild-type control. (b) At least three females were tested for each

mutant allele. (c) Embryos were examined around shield stage (~6 hpf; wild-type/total)

and at 24 hpf (wild-type/total). The few ‘non-wild-type’ embryos typically corresponded

to unfertilized. (d) Numbers obtained from multiple matings (typically two) of the

corresponding female.
