# Supplementary material for: Molecular genetics of maternally-controlled cell divisions
Source: PLoS Genet. 2020 Apr 8;16(4):e1008652. doi: 10.1371/journal.pgen.1008652 (PMC7179931; doi:10.1371/journal.pgen.1008652)
Supplement: S2 Table — (DOCX) [file pgen.1008652.s006.docx]

**S2 Table. Complementation crosses of *p10umal*/+ X *sa1624/sa1624***

| Genotype* | Cross | Wild-type embryos | Mutant Embryos** |
| --- | --- | --- | --- |
|  |  |  |  |
| *sa1624*/+ | 1 | 182 | 0 |
|  | 2 | 40 | 0 |
|  | 3 | 112 | 0 |
|  |  |  |  |
| *p10umal*/*sa1624* | 1 | 0 | 141 |
|  | 2 | 0 | 81 |
|  | 3 | 0 | 133 |

* females were genotyped using KASPar primers specific for *p10umal* and *sa1624* alleles

**mutant embryos appeared phenotypically normal at 2 hpf, failed to undergo epiboly (arrested

development) and lysed by 1 dpf
