# Supplementary material for: Molecular genetics of maternally-controlled cell divisions
Source: PLoS Genet. 2020 Apr 8;16(4):e1008652. doi: 10.1371/journal.pgen.1008652 (PMC7179931; doi:10.1371/journal.pgen.1008652)
Supplement: S3 Table — (DOCX) [file pgen.1008652.s007.docx]

**S3 Table.**

**Sequence of primer sets used in fine mapping**

| Primer | Sequence |
| --- | --- |
| sc2029-2F | 5’-GGCCCTATATGTGCCGTTTA-3’ |
| sc2029-2R | 5’-GATGCTTTAAATGAGCCGAAG-3’ |
| z42038-F | 5’-TGGCAGACCATCAATTTCAA-3’ |
| z42038-R  z11369-F  z11369-R  z7573-F  z7573-R  z9250-F  z9250-R  AL935188-2F  AL935188-2R  CU104756-4F  CU104756-4R | 5’-TCTCTCTCCTCCTCAGCCTG-3’  5’-GGGTAATATTGCACTTAGCCAGA-3’  5’-AACAAAAACCGTCTTGCCAC-3’  5’-TGTTGCACCATATTGTGGCT-3’  5’-AGACAAGAAAGGGGTCTGCA-3’  5’-TTCTTTCCAAACTGCAACCC-3’  5’-TCTCTCACTCGTGTGGGTGT-3  5’-TGGAGAAATGCTAATGTTCTGGTT-3’  5’-CAATCCCACTTTCAAAAATAAGC-3’  5’-TGCTAACCACTGAGCCATGA-3’  5’-TGCAGGGTGCAAATATGAGA-3’ |
| CU467110-F | 5’-AAATGGAGGACTTGTTCCAAAGAG-3’ |
| CU467110-R  BX004779-2F  BX004779-2R | 5’-CGTACATTTTGACCATGATTTACCA-3’  5’-GGAACAGACCACACTGAGAAGAGA-3’  5’-TTAATTGGAAGTCCCCATGAAGAT-3’ |
| BX510945-1F | 5’-TGCCAGTGGGTCAATGTAAA-3’ |
| BX510945-1R | 5’-AGGGTTAAGTGCGTGCATGT-3’ |
| BX511168-1F | 5’-AGGACGTCACCAATGTCCTC-3’ |
| BX511168-1R | 5’-CTTCACGATGCTTTCAGTGC-3’ |

‘F’= forward, ‘R’= reverse
